# LLY-507, a Cell-active, Potent, and Selective Inhibitor of Protein-lysine Methyltransferase SMYD2*[Fn FN2]

**DOI:** 10.1074/jbc.M114.626861

**Published:** 2015-03-30

**Authors:** Hannah Nguyen, Abdellah Allali-Hassani, Stephen Antonysamy, Shawn Chang, Lisa Hong Chen, Carmen Curtis, Spencer Emtage, Li Fan, Tarun Gheyi, Fengling Li, Shichong Liu, Joseph R. Martin, David Mendel, Jonathan B. Olsen, Laura Pelletier, Tatiana Shatseva, Song Wu, Feiyu Fred Zhang, Cheryl H. Arrowsmith, Peter J. Brown, Robert M. Campbell, Benjamin A. Garcia, Dalia Barsyte-Lovejoy, Mary Mader, Masoud Vedadi

**Affiliations:** From the ‡ Departments of Oncology Discovery, Structural Biology, Tailored Therapeutics, and Discovery Chemistry Research and Technologies, Eli Lilly and Company, Indianapolis, Indiana 46285,; § Structural Genomics Consortium, University of Toronto, 101 College Street, MaRS South Tower, 7th floor, Toronto, Ontario M5G 1L7, Canada,; ‖ Department of Medical Biophysics, University of Toronto and Princess Margaret Cancer Centre, 101 College Street, MaRS South Tower, Suite 707, Toronto, Ontario M5G 1L7, Canada, and; ¶ Department of Biochemistry and Biophysics, University of Pennsylvania, Philadelphia, Pennsylvania 19104

**Keywords:** cancer biology, crystal structure, enzyme inhibitor, epigenetics, protein methylation, SMYD2, chemical probe, methyltransferase

## Abstract

SMYD2 is a lysine methyltransferase that catalyzes the monomethylation of several protein substrates including p53. SMYD2 is overexpressed in a significant percentage of esophageal squamous primary carcinomas, and that overexpression correlates with poor patient survival. However, the mechanism(s) by which SMYD2 promotes oncogenesis is not understood. A small molecule probe for SMYD2 would allow for the pharmacological dissection of this biology. In this report, we disclose LLY-507, a cell-active, potent small molecule inhibitor of SMYD2. LLY-507 is >100-fold selective for SMYD2 over a broad range of methyltransferase and non-methyltransferase targets. A 1.63-Å resolution crystal structure of SMYD2 in complex with LLY-507 shows the inhibitor binding in the substrate peptide binding pocket. LLY-507 is active in cells as measured by reduction of SMYD2-induced monomethylation of p53 Lys^370^ at submicromolar concentrations. We used LLY-507 to further test other potential roles of SMYD2. Mass spectrometry-based proteomics showed that cellular global histone methylation levels were not significantly affected by SMYD2 inhibition with LLY-507, and subcellular fractionation studies indicate that SMYD2 is primarily cytoplasmic, suggesting that SMYD2 targets a very small subset of histones at specific chromatin loci and/or non-histone substrates. Breast and liver cancers were identified through *in silico* data mining as tumor types that display amplification and/or overexpression of SMYD2. LLY-507 inhibited the proliferation of several esophageal, liver, and breast cancer cell lines in a dose-dependent manner. These findings suggest that LLY-507 serves as a valuable chemical probe to aid in the dissection of SMYD2 function in cancer and other biological processes.

## Teaser

**Background:** SMYD2 is a methyltransferase whose role in cancer is poorly understood and is lacking cell-active chemical tools.

**Results:** We describe LLY-507, a small molecule inhibitor of SMYD2.

**Conclusion:** LLY-507 is potent, selective, cell-active, and binds SMYD2 in a high resolution co-crystal.

**Significance:** LLY-507 is a first-in-class cell-potent chemical probe that will be valuable in dissecting SMYD2 biology.

## Introduction

Protein-lysine methyltransferases represent one of several families of enzymes with critical roles in epigenetic regulation. Through the Su(var)3–9, enhancer of zeste, and trithorax (SET)^2^ domain, they catalyze the transfer of methyl groups from *S*-adenosyl-l-methionine to acceptor lysine residues on histone and/or non-histone protein substrates, impacting a variety of biological processes and disease states. Several protein-lysine methyltransferases are under pursuit as drug targets for cancer and/or other diseases (for reviews, see Refs. 1–3).

SMYD2 belongs to a five-member (SMYD1–5) human SET and MYND (SMYD) domain-containing subfamily of protein-lysine methyltransferases. The SMYD enzymes display a distinct protein architecture in which the SET sequence is split by a zinc finger containing the myeloid translocation protein-8, Nervy, and DEAF-1 (MYND) motif followed by a cysteine-rich post-SET domain. All members of the SMYD family show high identity and similarity in the SET and MYND domains, although the homology is much less conserved in other subdomains of the SMYD family (4).

The SMYD proteins have distinct substrate specificities and play diverse roles in muscle development and/or cancer. SMYD1 is a heart and skeletal muscle-specific histone H3K4 methyltransferase involved in mouse heart development and zebrafish myogenesis (5, 6). SMYD3 targets both histone (histone H3K4 and histone H4K5) and non-histone substrates (VEGFR1, estrogen receptor α, and MAP3K2), is overexpressed in cancer and implicated in the tumorigenesis of Ras-driven tumors, and plays a role in skeletal atrophy through recruitment of bromodomain protein Brd4 and regulation of c-Met and myostatin gene expression (7–10). Disruption of one SMYD4 allele in nontumorigenic mouse mammary epithelial cells results in tumorigenesis that is reversible with SMYD4 re-expression, suggesting that SMYD4 may be a potential tumor suppressor (11). SMYD5 trimethylates lysine 20 on histone H4 on a specific subset of Toll-like receptor 4 genes and represses their expression in association with nuclear co-repressor complexes, suggesting a role for this methyltransferase in inflammation (12).

Like SMYD3, SMYD2 also targets a number of histone and non-histone substrates including histones H3, H4, and H2B (4, 13, 14); p53 (15); retinoblastoma (Rb; Refs. 16 and 17); HSP90 (18, 19); estrogen receptor α (20, 21); and poly(ADP-ribose) polymerase (22) and is implicated in both cancer and muscle development. Several SMYD2-peptide substrate co-crystal structures have been solved (21, 23–26). The majority of SMYD2 non-histone substrates play roles in cancer-related processes. It is thought that mono-methylation by SMYD2 of lysine Lys^370^ on p53 creates a steric blockage that impedes p53 from binding its target gene promoters, and knockdown of SMYD2 prevents DNA damage-induced, p53-dependent apoptosis (15). SMYD2-mediated methylation of Rb has been reported on lysines 810 (16) and 860 (17); lysine 860 methylation on Rb by SMYD2 is thought to result in the repression of specific Rb/E2F genes through the recruitment of the lysine methyl-binding protein L3MBTL1 (17), whereas lysine 810 methylation on Rb by SMYD2 enhances Rb phosphorylation on serines 807/811 and subsequent E2F transcriptional activity and cell cycle progression (16). Under estrogen-depleted conditions, estrogen receptor α is prevented from being recruited to its target gene promoters when methylated on Lys^266^ by SMYD2 (20). In addition to targeting cancer cell regulatory proteins, the SMYD2 gene resides in 1q32-q41, a chromosomal region found to be frequently amplified in esophageal squamous cell carcinoma (ESCC) and hepatocellular carcinoma (HCC) (27, 28). Overexpression of SMYD2 is found in 75 and 95% of tested ESCC (27) and pediatric acute lymphoblastic leukemia (29) primary tumors, respectively, and this overexpression is correlated with poor patient survival. Furthermore, knockdown of SMYD2 inhibits and overexpression of SMYD2 promotes ESCC cell proliferation (27).

The oncogenic nature of its target substrates and its involvement in ESCC and pediatric acute lymphoblastic leukemia suggest that SMYD2 plays a potential role in oncogenesis. To validate and further investigate the mechanisms by which SMYD2 is implicated in cancer and other biological processes, 3-cyano-5-{2-[4-[2-(3-methylindol-1-yl)ethyl]piperazin-1-yl]-phenyl}-*N*-[(3-pyrrolidin-1-yl)propyl]benzamide (LLY-507), a potent cell-permeable and selective small molecule inhibitor of SMYD2, was developed. In this report, we disclose the biochemical and cellular characterization of LLY-507 as well as the crystal structure of SMYD2 in complex with LLY-507.

## Experimental Procedures

### 

#### 

##### Chemical Synthesis of LLY-507

LLY-507 was prepared by methods described in the literature utilizing 3-methylindole, methyl 3-cyanobenzoate, 3-(pyrrolidin-1-yl)propan-1-amine, and 1-bromo-2-iodobenzene as the key building blocks (detailed in the supplemental methods). LLY-507 is available through the SGC (Structural Genomics Consortium) until available commercially. It can be found under “chemical probes.”

##### SMYD2 Biochemical Assay

Methyltransferase activity assays for SMYD2 were performed by monitoring the incorporation of tritium-labeled methyl groups to a peptide corresponding to residues 361–380 of p53 or residues 1–24 of H4 using the scintillation proximity assay. The enzymatic reactions with p53 substrate were performed at 23 °C with 1-h incubation of a 20-μl reaction mixture in 20 mm Tris-HCl, pH 9, 5 mm DTT, 0.01% Triton X-100, 0.5 μm *S*-[methyl-^3^H]adenosyl-l-methionine (PerkinElmer Life Sciences), 3 μm p53(361–380) peptide, 30 nm SMYD2, and 0.3 nm to 5 μm LLY-507. The enzymatic reactions with H4 substrate were performed at 23 °C with 1-h incubation of a 20-μl reaction mixture in 50 mm Tris, pH 9, 2 mm DTT, 0.01% Triton X-100, 0.25 μm *S*-[methyl-^3^H]adenosyl-l-methionine, 1 μm H4(1–24) peptide, 30 nm SMYD2, and 3 nm to 3 μm LLY-507. To quench reactions, 20 μl of 7.5 m guanidine hydrochloride followed by 180 μl of buffer (20 mm Tris, pH 8.0) was added. After mixing, the quenched reaction mixture was transferred to a 384-well streptavidin-coated FlashPlate® (PerkinElmer Life Sciences) followed by incubation for 2 h. Counts per minute (cpm) was measured using the TopCount® plate reader (PerkinElmer Life Sciences). cpm in the absence of compound for each data set was defined as 100% activity. cpm in each data set in the absence of enzyme was defined as background (0%). All enzymatic reactions were performed in triplicate, and IC_50_ values were determined by fitting the data to the four parameter logistic equation using SigmaPlot software.

##### Selectivity Assays

The effect of LLY-507 on the methyltransferase activity of G9a, EHMT1, SUV39H2, SETDB1, SETD7, SETD8, SUV420H1, SUV420H2, PRMT1, PRMT3, PRMT6, PRMT8, PRDM9, SETD2, SMYD2, MLL1, MLL3, EZH1, EZH2, and PRMT5 complexes as well as DNMT1 was assessed using the scintillation proximity assay method as described above at substrate concentrations close to the *K_m_* values for each enzyme. Assay conditions are summarized in supplemental Table 1. Three concentrations (1, 10, and 50 μm) of LLY-507 were used in all selectivity assays. For DNMT1, the double-stranded DNA substrate was prepared by annealing two complementary strands (forward strand, biotin-GAGCCCGTAAGCCCGTTCAGGTCG; reverse strand, CGACCTGAACGGGCTTACGGGCTC) synthesized by Eurofins MWG Operon. For DOT1L, NSD1, NSD2, NSD3, and SMYD3, a filter-based assay was used. In this assay, 20 μl of reaction mixture was incubated at 23 °C for 1 h followed by addition and mixing of 100 μl of % trichloroacetic acid (TCA). Reaction mixtures were transferred to filter plates (Millipore, catalog number MSFBN6B10). Plates were centrifuged at 2000 rpm in the Allegra® X-15R (Beckman Coulter) for 2 min followed by two additional 10% TCA washes, one ethanol wash (180 μl), and centrifugation. Plates were dried, and 70 μl of MicroScint^TM^-O (PerkinElmer Life Sciences, catalog number 6013611) was added. cpm was measured using a TopCount plate reader.

##### Thermal Denaturation Fluorometry

SMYD2 protein unfolding was monitored on a 384-well Roche Lightcycler480 II^TM^ RT-PCR machine using SYPRO® Orange as a reporter dye (Invitrogen, S6651). Excitation and emission filters were set at 465 and 580 nm, respectively; the temperature was continuously ramped from 25 to 95 °C at a rate of 0.11°C/s. The reaction mixture (6 μl/well) contained 3.9 μm SMYD2 protein and 100 μm compound in 20 mm Tris, pH 8.0, 150 mm NaCl, 10% glycerol, 2 mm tris(2-carboxyethyl)phosphine (TCEP), and 1% DMSO buffer. Triplicates were run along with protein without compound as a negative control.

##### Crystallography

SMYD2 crystals were grown by vapor diffusion in sitting drop trays at 8 °C. Protein at 12.6 mg/ml in 20 mm Tris, pH 9.0, 150 mm NaCl, and 2 mm DTT was mixed with an equal amount of reservoir solution containing 100 mm Tris-HCl, pH 8.6, 14% PEG 20,000, and 200 mm sodium chloride and equilibrated for a few days. Crystals grew to 100 μm within 4–5 days. Crystals were soaked overnight in a solution comprising 5 mm LLY-507, 100 mm Tris, pH 8.6, 200 mm NaCl, 18% PEG 20,000, and 5 mm tris(2-carboxyethyl)phosphine (TCEP). The crystals were then transferred to a cryosolution supplemented with 25% glycerol and flash frozen. Data were collected on the Lilly Research Laboratories Collaborative Access Team beam-line 31-ID at the Advanced Photon Source at Argonne National Laboratory, Argonne, IL. The crystals diffracted to 1.63 Å, belonged to space group P21212, and contained one molecule of SMYD2 per asymmetric unit. The data were processed, and the structure was determined by molecular replacement using the CCP4 program suite (Collaborative Computational Project (31)) with an internal structure of SMYD2 as a search model. The structure was refined to *R*-factors of *R*_working_= 15.1% and *R*_free_= 20.0%. The final structure had good geometry with 93% of the residues in the most favored regions of the Ramachandran map and no residues in disallowed regions. Additional crystallography details are included in Fig. 2*D*.

##### Cell-based p53 Methylation Assays

To examine the methylation status of p53 in HEK293 cells treated with LLY-507 by Western blotting, 2 × 10^5^ cells were seeded in 6-well plates in triplicate and co-transfected with FLAG-tagged p53 and FLAG-tagged SMYD2 using Lipofectamine® 2000 (Invitrogen). The day after transfection, cells were treated with 0–2.5 μm LLY-507 for 28 h, then collected, and lysed in radioimmunoprecipitation assay (RIPA) buffer. Cell lysates were subject to 10% SDS-PAGE and transferred to a PVDF membrane. Each membrane was blocked in Odyssey Blocking Buffer (LI-COR Biosciences) and incubated with monoclonal anti-p53 (D01) antibody (Abcam, ab1101) or rabbit anti-p53 Lys^370^me1 antibody (a kind gift from Dr. Shelley Berger) followed by detection with IRDye®680RD goat anti-mouse IgG (LI-COR Biosciences) and IRDye800CW goat anti-rabbit IgG (LI-COR Biosciences) on the Odyssey Imager (LI-COR Biosciences). To determine the effect of LLY-507 on SMYD2-induced methylation in U2OS cells using a cell-based ELISA, cells were transfected with FLAG-tagged SMYD2 using Lipofectamine 2000. 4–8 h later, cells were treated with 0–25 μm compound for 15 h. Cells were then fixed with Prefer (Anatech), permeabilized in 0.1% Triton X-100, blocked using Odyssey Blocking Buffer, and incubated with a custom-made affinity-purified polyclonal anti-p53 Lys^370^me1 antibody (Lampire) followed by detection with IRDye800CW goat anti-rabbit IgG on the Odyssey Imager. To determine the effect of LLY-507 on the methylation of p53 in KYSE-150 cells stably overexpressing FLAG-tagged SMYD2 by sandwich ELISA, 1 × 10^6^ cells were seeded in 6-well plates and the following day treated with 0–5 μm LLY-507. 24 h later, cells were lysed with 200 μl of MSD Tris Lysis Buffer^TM^ (Meso Scale Discovery). A MULTI-ARRAY^TM^ 96-well plate (Meso Scale Discovery, catalog number L15XA-3) was precoated with 2 μg/ml custom-made methyl-p53 antibody (Lampire) in PBS overnight at 4 °C. 150 μl/well MSD Blocking Solution A^TM^ (final concentration of 3%) was added followed by shaking at room temperature for 1 h. The plate was washed three times with 1× MSD Tris Wash Buffer^TM^ (Meso Scale Discovery). 0.5 μg of cell lysate in 25 μl/well MSD Tris Lysis Buffer was added, followed by shaking overnight at 4 °C. The plate was washed three times with 1× MSD Tris Wash Buffer. SULFO-TAG^TM^ total p53 detection antibody was added to the plate at a final concentration of 1.5 mg/ml in Antibody Dilution Buffer (Meso Scale Discovery) followed by shaking for 1 h at room temperature. Following a final wash three times with 1× MSD Tris Wash Buffer, 150 μl/well 1× MSD Read Buffer^TM^ (Meso Scale Discovery) was added, and plates were read on an MSD SECTOR^TM^ Imager 6000 instrument (Meso Scale Discovery).

##### Mass Spectrometry Proteomics of Cell-derived Histones

Acid-extracted histone samples were chemically derivatized using propionic anhydride and digested with trypsin for 6 h at 37 °C. The digested peptides were treated with an additional round of propionylation and desalted using a C_18_ extracted mini disk (Empore 3M). Approximately 1 μg of each sample was loaded via an autosampler (Eksigent NanoLC.Ultra 2D Plus) onto a homemade precolumn (3-μm particle size, 120-Å pore size) at a flow rate of 2 μl/min. Peptides were chromatographically resolved via a 75-μm reversed phase analytical column packed with C_18_-AQ resin (1.9-μm particle size, 120-Å pore size) using a 77-min 2–98% solvent B gradient (solvent A, 0.1% formic acid; solvent B, 100% acetonitrile) at a flow rate of 250 nl/min. The electrosprayed peptides were detected by an Orbitrap Elite^TM^ mass spectrometer (Thermo Fisher Scientific) with a resolution of 120,000 for full MS spectrum followed by MS/MS spectra obtained in the ion trap. The relative abundance of each modification, expressed as a percentage on a histone peptide sequence, was quantified by analyzing its MS and MS/MS spectra via Epiquant, an in-house software.

##### Subcellular Fractionation and Western Blots

5 × 10^6^ KYSE-150 cells or KYSE-150 cells stably overexpressing SMYD2 were harvested and separated into subcellular fractions using the Subcellular Protein Fractionation kit for cultured cells (Thermo Scientific) according to the manufacturer's instructions. Lysates were reduced, boiled, subjected to 4–20% SDS-PAGE, and transferred to nitrocellulose membranes. Membranes were blocked with 5% Difco skim milk (BD Biosciences) and incubated for 2 h at room temperature or overnight at 4 °C with the following antibodies: custom-made affinity-purified polyclonal anti-p53 Lys^370^me1 antibody (Lampire), monoclonal anti-p53 (D01) antibody, anti-SMYD2 (Cell Signaling Technology), anti-α-tubulin (Sigma), anti-RNA polymerase II (Cell Signaling Technology), and anti-H2AX (Cell Signaling Technology). Each membrane was then incubated with ECL anti-rabbit or anti-mouse IgG HRP-linked whole antibody (GE Healthcare) followed by detection with SuperSignal West Femto Maximum Sensitivity Substrate (Thermo Scientific) on a ChemiDoc^TM^ XRS system (Bio-Rad). Primary tumor lysates in RIPA buffer were obtained from Crownbio Bioscience. Cancer cell lines were obtained from the German Collection of Microorganisms and Cell Cultures GmbH (DSMZ), the Korean Cell Line Bank, the Japanese Collection of Research Bio-Resources, or the American Type Culture Collection (ATCC). Tumor cells were lysed in buffer containing 50 mm Tris-HCl, pH 7.5, 500 mm NaCl, 1% Nonidet P-40, 0.25% sodium deoxycholate, 20 mm NaF, protease inhibitor mixture (Roche Applied Science), and phosphatase inhibitor mixtures I and II (Sigma). Tumor or cell lysates were boiled, subjected to 4–20% SDS-PAGE, and transferred to nitrocellulose membrane. Each membrane was blocked in Odyssey Blocking Buffer and incubated with custom-made mouse monoclonal anti-SMYD2 or mouse monoclonal anti-β-actin (Sigma) antibody followed by detection with IRDye680RD goat anti-mouse IgG on the Odyssey Imager.

##### Proliferation Assays

To examine the effect of LLY-507 on tumor cell proliferation, cells were seeded in 96-well plates overnight at various densities optimized for 3 to 4- or 7-day growth and then treated with 0–20 μm LLY-507 for 3–7 days. Cell viability was measured using CellTiter-Glo^TM^ (Promega) according to the manufacturer's instructions.

## Results

### 

#### 

##### Chemical Structure and Biochemical Characterization of LLY-507

**FIGURE 1.**
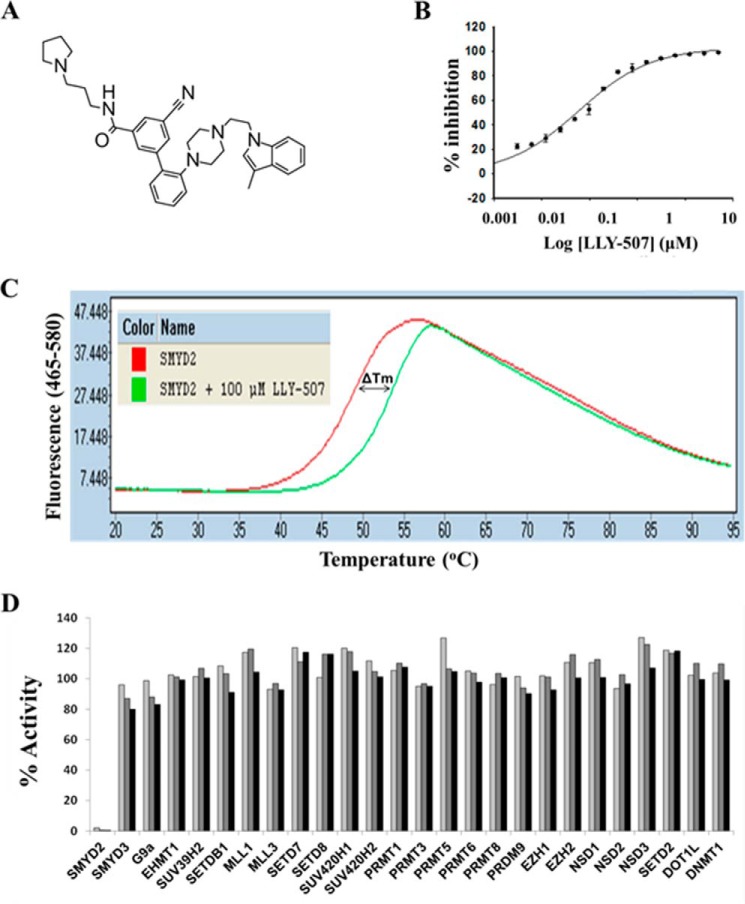
**Chemical structure and biochemical profile of LLY-507.** *A*, chemical structure of LLY-507. *B*, effect of LLY-507 on the methyltransferase activity of SMYD2 using p53(361–380) peptide as a substrate (IC_50_ < 0.015 μm). *Error bars* represent S.D. *C*, thermostability melting curves of SMYD2 in the absence (*red*) or presence (*green*) of 100 μm LLY-507. *D*, effect of LLY-507 at 1 (*light gray*), 10 (*dark gray*), and 50 μm (*black*) against SMYD2 and 25 other protein or DNA methyltransferases.

LLY-507 was designed by applying principles for peptide-competitive inhibitors reported by Ferguson *et al.* (25). The group found that a polar group such as pyrrolidine could extend into the substrate lysine channel and that affinity was increased by additionally occupying two lipophilic pockets, a primary pocket along the peptide backbone binding groove and a secondary pocket remote from the peptide substrate interactions. More recently, they demonstrated that similar concepts were successfully applied to optimizing an additional screening library hit. Leveraging these observations while remaining in property space of polar surface area (<80) and lipophilicity (*c*log*P* >5) that enabled cell permeability while retaining enzymatic potency yielded compound LLY-507 (Fig. 1*A*).

The ability of LLY-507 to inhibit the catalytic function of SMYD2 was tested by scintillation proximity assay using peptides derived from a previously described p53 SMYD2 substrate (24). LLY-507 potently inhibited the ability of SMYD2 to methylate p53 peptide with an IC_50_ <15 nm (Fig. 1*B*). Binding of LLY-507 to SMYD2 was further confirmed using differential scanning fluorometry (30). The presence of 100 μm LLY-507 increased the thermal stability of SMYD2 (*T_m_* = 49 °C) by 5 °C (*T_m_* = 54 °C), and the stabilization effect was concentration-dependent (Fig. 1*C*). SMYD2 was the only one of 25 tested methyltransferases including related family members SMYD3, SUV420H1, and SUV420H2 that was potently inhibited (>100-fold difference) by LLY-507 (Fig. 1*D*). The compound was further profiled against 454 kinases, 36 G protein-coupled receptors, 14 nuclear hormone receptors, and three cytochrome P450 enzyme targets (supplemental Tables 2–4). The only inhibitory activity that was noted (at 98% at 10 μm) targeted the calcium mobilization antagonist mode of hADRα1A, a member of the G protein-coupled receptor family involved in epinephrine signaling.

##### High Resolution Co-complex Structure of SMYD2 Bound to LLY-507

**FIGURE 2.**
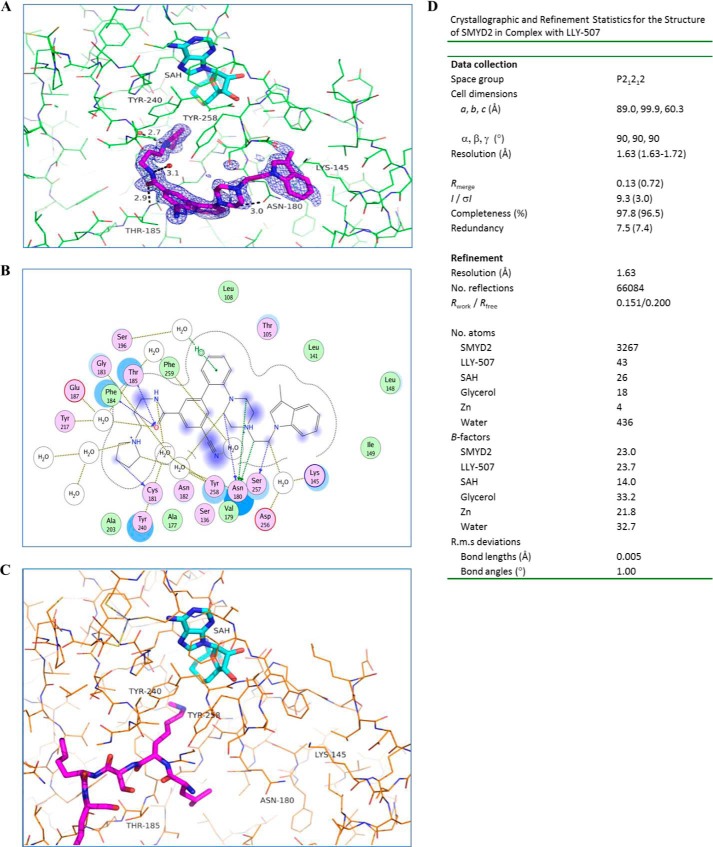
**Crystal structure of SMYD2 in complex with LLY-507.** *A*, SMYD2 is depicted as *thin sticks* in *green*, whereas the bound LLY-507 and *S*-adenosyl-l-homocysteine (*SAH*) are depicted as *thick sticks* in *magenta* and *cyan*, respectively. The *F_o_* − *F_c_* omit map electron density of LLY-507 in *blue* is contoured at 3σ. *B*, plot of SMYD2 interactions with LLY-507. *C*, structure of SMYD2 in complex with mono-methylated p53 peptide (Ref. 25; Protein Data Bank code 3S7D) with SMYD2 depicted as *thin sticks* in *orange*, and *S*-adenosyl-l-homocysteine and p53 peptide depicted as *thick sticks* in *cyan* and *magenta*, respectively. *D*, crystallographic and refinement statistics for the structure of SMYD2 in complex with LLY-507. *R.m.s.*, root mean square.

The structure of SMYD2 bound to LLY-507 was determined to a resolution of 1.63 Å (Fig. 2, *A*, *B*, and *D*), the coordinates of which have been deposited in the RCSB Protein Data Bank, under code 4WUY. SMYD2 in complex with LLY-507 adopts a structure very similar to what has been observed previously in structures with cofactor and cofactor analogs as well as in ternary complex structures with unmethylated and monomethylated substrate peptides (Fig. 2*C* and Refs. 23–26). LLY-507 binds in the substrate peptide binding site of SMYD2 with the pyrrolidine group ensconced in the lysine pocket formed by the side chains of Phe^184^, Tyr^240^, and Tyr^258^. The nitrogen atom of the pyrrolidine makes a hydrogen bond to an ordered water molecule, whereas the oxygen and nitrogen atoms of the methylene amide linker form hydrogen bonds to the main chain amide nitrogen of Thr^185^ and a bound water molecule, respectively. The benzonitrile group makes a C–H···O hydrogen bond to the main chain carbonyl of Gly^183^ with the nitrile group oriented toward the solvent. The piperazine packs against Asn^180^, and makes C–H···O hydrogen bonds to the Asn^180^ side chain. The indole stacks between the side chains of Lys^145^ and Asn^180^. The phenyl linking the benzonitrile to the piperazine sits in a pocket formed by the side chains of Thr^105^, Thr^145^, Asn^180^, and Ser^196^ as well as the backbone amide units of Val^179^, Gly^183^, and Phe^184^. This structure shows that LLY-507 binds to the substrate channel of SMYD2 and supports the selectivity of inhibition of LLY-507 for SMYD2.

##### LLY-507 Inhibits SMYD2-mediated Methylation of p53 Lys^370^ in Cells

**FIGURE 3.**
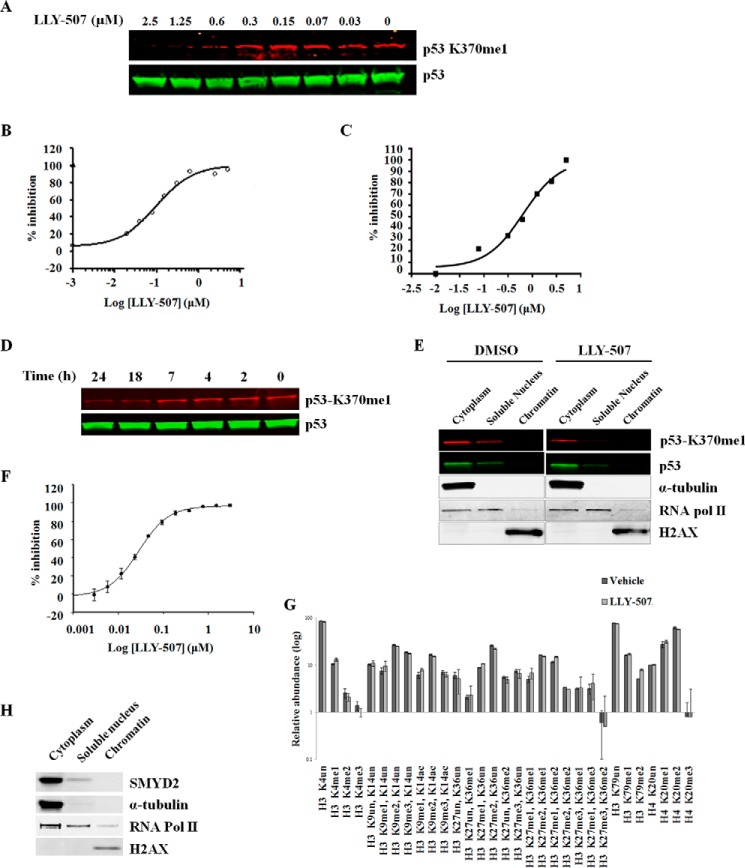
**LLY-507 inhibits SMYD2-mediated methylation of p53 Lys^370^ in cells.** *A*, Western blot showing concentration-dependent inhibition of p53 Lys^370^me1 following treatment with 0–2.5 μm LLY-507 in HEK293 cells transiently transfected with FLAG-SMYD2 and FLAG-p53 (IC_50_ < 1 μm). *B*, cell-based ELISA for p53 Lys^370^me1 of U2OS cells transfected with SMYD2 following treatment with LLY-507 for 15 h (IC_50_ = 0.6 μm). *C*, concentration-dependent inhibition of p53 Lys^370^me1 by LLY-507 in KYSE-150 cells stably expressing SMYD2 as measured by a Meso Scale Discovery sandwich ELISA (IC_50_ = 0.6 μm). *D*, Western blot showing a time-dependent decrease in p53 Lys^370^ mono-methylation with 5 μm LLY-507 treatment of KYSE-150 cells stably expressing FLAG-SMYD2. *E*, Western blot examining the protein levels of p53-Lys^370^me1 and p53 as well as α-tubulin, RNA polymerase (*pol*) II, and histone H2AX positive controls for cytoplasmic, soluble nuclear, and chromatin subcellular fractions, respectively, in KYSE-150 cells stably expressing FLAG-SMYD2. *F*, effect of LLY-507 on SMYD2 biochemical activity using histone H4(1–24) peptide as substrate (IC_50_ = 31 nm). *G*, relative intensities of methylated histone H3 or H4 peptides in SMYD2-overexpressing KYSE-150 cells treated with vehicle or 5 μm LLY-507, identified and quantified using mass spectrometry. A full list of histone H3 and H4 peptides and their intensities is found in supplemental Table 5. *H*, Western blot examining the protein levels of endogenous SMYD2 as well as α-tubulin, RNA polymerase (*Pol*) II, and histone H2AX positive controls for KYSE-150 cytoplasmic, soluble nuclear, and chromatin subcellular fractions, respectively. *un*, un-methylated; *me1*, mono-methylated; *me2*, di-methylated; *me3*, tri-methylated; *ac*, acetylated.

The methylation of p53 by SMYD2 has been observed in cells by several groups (15, 24, 25). LLY-507 was tested for its ability to inhibit SMYD2 in cells through its effects on cellular p53 methylation using three independent methods. First, analogous to the system described in Huang *et al.* (15) and using the antibody from the Berger laboratory, Western blot analysis for mono-methylated Lys^370^ of p53 was performed on lysates derived from LLY-507-treated HEK-293 cells transiently co-transfected with FLAG-tagged SMYD2 and FLAG-tagged p53. As shown in Fig. 3*A*, LLY-507 incurred a concentration-dependent decrease in levels of p53 Lys^370^me1 with an IC_50_ of less than 1 μm. Second, similar to the assay described in Ferguson *et al.* (25), LLY-507 was assessed for its ability to inhibit robust SMYD2 p53 methylation induction. This was demonstrated by transient transfection of FLAG-tagged SMYD2 in U2OS cells using a cell-based ELISA and custom-made purified anti-mono-methyl p53 Lys^370^ antibody. LLY-507 inhibited SMYD2-induced p53 methylation in U2OS cells with an IC_50_ of 0.6 μm (Fig. 3*B*). To ensure that the methylation that was being measured was specific to p53, a mono-methyl-Lys^370^ p53/total p53 sandwich ELISA was also developed. FLAG-tagged SMYD2 stable cell lines were established using KYSE-150 cells, an ESCC cell line showing amplification and overexpression of SMYD2 (27), and tested for the effects of LLY-507 on the levels of mono-methyl-Lys^370^ p53. LLY-507 reduced levels of mono-methyl-Lys^370^ p53 in a dose-dependent manner at the same potency as in the U2OS cell-based ELISA with an IC_50_ of 0.6 μm (Fig. 3*C*). These results show that LLY-507 exhibits submicromolar potency against SMYD2 in cells, making it the first selective and cell-active small molecule inhibitor of SMYD2.

One of the benefits of small molecule inhibitors over genetic loss-of-function approaches is that they directly and rapidly inhibit the activity of their protein target, rather than depend on the depletion first of mRNA followed by protein. We took advantage of LLY-507 to determine the time frame at which p53 Lys^370^ methylation is affected in response to inhibition of SMYD2 catalytic function. As shown in Fig. 3*D*, significant reductions in p53 Lys^370^ methylation occurred by 18 h following LLY-507 treatment of SMYD2-overexpressing KYSE-150 cells. The methylation on Lys^370^ was affected on both cytoplasmic (by 50%) and nuclear (by 25%) forms of p53, whereas total levels of p53 were not significantly affected by LLY-507 treatment (Fig. 3*E*).

**FIGURE 4.**
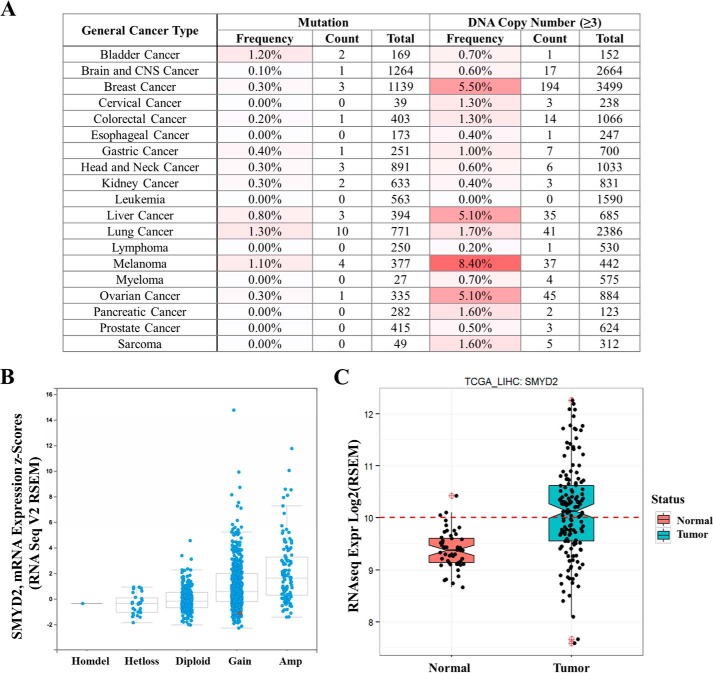
**Bioinformatics analysis of SMYD2 in cancer.** *A*, frequency of SMYD2 mutations and increased DNA copy number in various tumor types (Oncomine). *B*, The Cancer Genome Atlas (*TCGA*) expression analysis of SMYD2 in breast cancer. *C*, The Cancer Genome Atlas expression analysis of SMYD2 in liver tumors compared with normal samples. *RNA-Seq*, RNA sequencing; *Expr*, expression; *Amp*, amplified; *Homdel*, homozygous deletion; *Hetloss*, loss of heterozygosity.

At the biochemical level, recombinant SMYD2 is also able to methylate histone H3 and H4 peptides and proteins (4, 13, 24), and in this study, LLY-507 was able to potently inhibit the methylation of H4 peptide by SMYD2 enzyme with an IC_50_ of 31 nm (Fig. 4*D*). At the cellular level, however, contradicting observations have been reported. Although two reports demonstrated that transient overexpression of SMYD2 in cells induces the methylation of histone H3K4 and/or histone H3K36 (4, 13), another study showed that SMYD2-deficient murine cardiomyocytes do not exhibit any effect on the methylation of these two residues in SMYD2-deficient cells compared with their wild-type counterparts (32). All studies used antibody-based methods to measure histone methylation levels in cells. To circumvent potential artifacts commonly associated with methylation-specific antibodies, the effects of SMYD2 inhibition by LLY-507 on histone post-translational modifications in cells was assessed using mass-spectrometry based proteomics. LLY-507-treated KYSE-150 cells overexpressing SMYD2 did not show any significant change in the lysine methylation of histone H3 or H4 compared with untreated cells (Fig. 3*E* and supplemental Table 5). Consistent with these findings, subcellular fractionation studies were performed and revealed that endogenous SMYD2 protein was primarily localized in the cytoplasm of KYSE-150 cells (Fig. 3*F*). These results suggest that either SMYD2 does not target histones in cells or that SMYD2-mediated methylation of histones occurs only locally on a small percentage of gene loci.

##### Bioinformatics Analysis of SMYD2 Expression in Primary Tumors

To test the role of SMYD2 catalytic function in tumor cell proliferation using LLY-507, we sought to identify tumor types in which SMYD2 could function as a potential driver by means of amplification and/or overexpression. Komatsu *et al.* (27) demonstrated that SMYD2 is overexpressed in primary ESCC tumors. To see whether SMYD2 is altered at the genomic or expression level in cancers in addition to ESCC, bioinformatics analysis of SMYD2 in primary tumors was performed using Oncomine and The Cancer Genome Atlas databases. Oncomine analysis of primary tumors from 14 different cancer types showed that SMYD2 is not frequently mutated in cancer (Fig. 4*A*, *left*). However, using a DNA copy number threshold of 3 and above, SMYD2 is potentially amplified in 5–8% cases of breast, liver, melanoma, or ovarian cancer (Fig. 4*A*, *right*). The Cancer Genome Atlas analysis was also performed to identify tumor types showing overexpression of SMYD2 at the transcript level. As shown in Fig. 4*B*, ∼28% of breast tumors show both SMYD2 amplification as well as higher gene expression compared with diploid samples. Liver tumors also show a significant increase in SMYD2 expression compared with normal samples (Fig. 4*C*). These results show that in addition to ESCC SMYD2 is also amplified and/or overexpressed in breast and liver primary tumors.

##### SMYD2 Protein Expression Levels in Esophageal, Breast, and Liver Primary Tumors and/or Cell Lines

**FIGURE 5.**
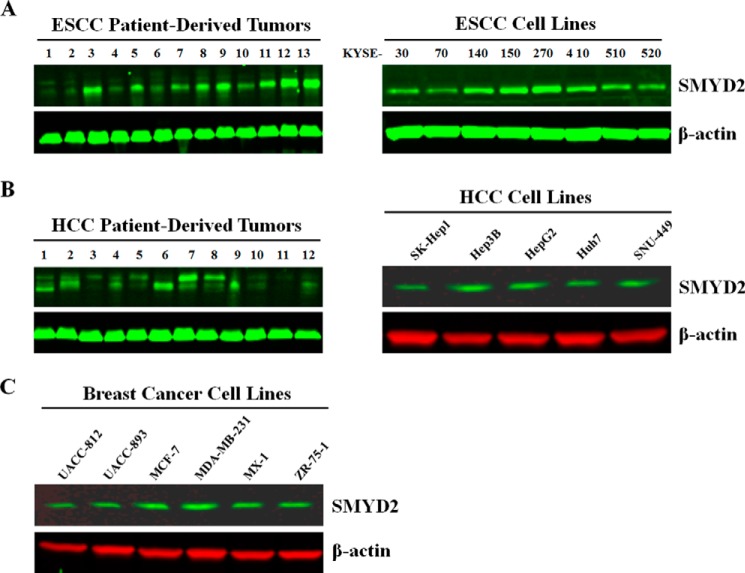
**SMYD2 protein expression levels in ESCC, HCC, and breast cancer primary tumors and cell lines.** Western blots show SMYD2 protein levels in ESCC primary tumors and cell lines (*A*), HCC primary tumors and cell lines (*B*), and breast cancer cell lines (*C*).

To corroborate the bioinformatics data, Western blotting for SMYD2 protein expression was performed on panels of ESCC, breast, and HCC primary tumors (where available) or cell lines. As shown in Fig. 5*A* (*left*), SMYD2 protein was expressed in a significant number (11 of 13; 85%) of ESCC primary tumors. Furthermore, SMYD2 was also expressed in all ESCC lines tested with levels being the highest in KYSE-150 and KYSE-270 (Fig. 5*A*, *right*). These results are consistent with those of Komatsu *et al.* (27). SMYD2 protein was also detected in nine of 12 (75%) HCC patient-derived tumors and all HCC cell lines (Fig. 5*B*), although in some of the primary tumor samples, SMYD2 appeared at a slightly higher molecular weight, the explanation of which is not known. Primary breast cancer tumors were not available, but all breast cancer cell lines tested expressed SMYD2 to a similar degree (Fig. 5*C*). These results are consistent with the results from the bioinformatics analysis of SMYD2 and show that ESCC, HCC, and breast cancer tumor samples and cell lines do indeed express SMYD2 at the protein level.

##### LLY-507 Inhibits Tumor Cell Proliferation

**FIGURE 6.**
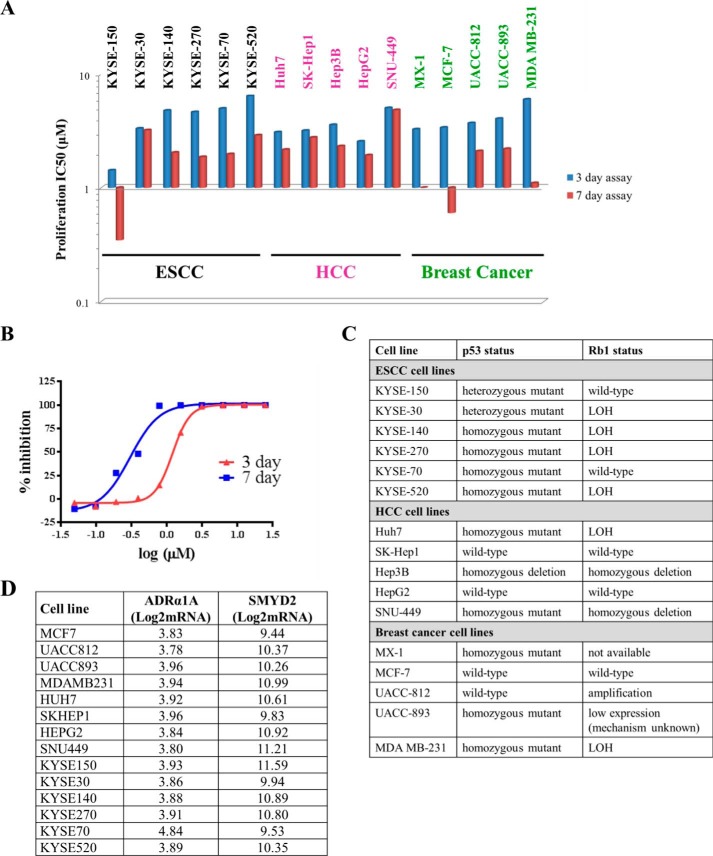
**LLY-507 inhibits the proliferation of several ESCC, HCC, and breast cancer cell lines.** *A*, effect of 3- or 7-day treatment with LLY-507 on the proliferation of ESCC, HCC, and breast cancer cell lines. Cells were treated for either 3–4 or 7 days with compound, and cell viability was then measured using CellTiter-Glo. *B*, concentration-response curves depicting effect of LLY-507 on 3- and 7-day growth of KYSE-150 cells. *C*, summary of the status of p53 or Rb in the ESCC, HCC, and breast cancer cell lines tested for the antiproliferative effects of LLY-507 derived from mining of the Cancer Cell Line Encyclopedia. *D*, mRNA expression analysis of hADR-α1A and SMYD2 in cell lines used in this study derived from mining of the Cancer Cell Line Encyclopedia. *LOH*, loss of heterozygosity.

*In silico* analyses and SMYD2 protein expression patterns in ESCC, HCC, and breast cancer primary tumors and/or cell lines suggest that these tumor types may be potentially driven by SMYD2. The antiproliferative effects of SMYD2 inhibition in the three tumor types were explored using LLY-507. In addition to the standard 3 to 4-day format, a 7-day duration format similar to those used to test inhibitors of confirmed epigenetic targets EZH2, DOT1L, and G9a was also performed to capture potential SMYD2-driven epigenetic antiproliferative effects. Results of the proliferation assays are graphically summarized in Fig. 6*A*, and an example concentration-response curve is shown in Fig. 6*B*. Cell lines from all three tumor cell panels responded to LLY-507 with IC_50_ values ranging from 1.5 to 6 μm in the standard assay and from 0.3 to 3.2 μm in the longer term assay. KYSE-150, among the cell lines showing the highest expression of SMYD2, was the most sensitive of the cell lines in the ESCC panel. All HCC cell lines tested showed similar IC_50_ values for LLY-507 and did not show increased sensitivity to LLY-507 with time, suggesting that SMYD2-mediated growth in liver cancer is likely not directly mediated by epigenetic mechanisms. Within the breast cancer cell line panel, MDA MB-231 cells were distinct as they demonstrated a >5-fold increase in sensitivity to LLY-507 at 7 days compared with 3 to 4-day treatment, suggesting that proliferation of this cell line may be at least in part mediated by SMYD2-dependent epigenetic mechanisms.

Analysis of the relationship between SMYD2 expression and LLY-507 IC_50_ shows that sensitivity to LLY-507 is not dependent on levels of SMYD2 protein. As SMYD2 substrates p53 and Rb are often found to be inactivated in cancer, it was hypothesized that if the methylation of these substrates by SMYD2 played a role in SMYD2-mediated proliferation then heightened sensitivity to LLY-507 would occur in cell lines with wild-type p53 or Rb. Analysis of the relationship between p53 or Rb mutation status mined from the Cancer Cell Line Encyclopedia (summarized in Fig. 6*C* and see Refs. 33 and 34) and the antiproliferative effect of LLY-507 on the tested cell lines revealed that there is no correlation between the presence of wild-type p53 and/or Rb and sensitivity to LLY-507. These results suggest that methylation of p53 or Rb by SMYD2 is not the principal driver of SMYD2-mediated cancer cell growth and that other SMYD2 substrates or a second genetic or epigenetic driver may be involved. To investigate the potential implications of the observed off-target activity of LLY-507 on hADR-α1A, we performed an *in silico* search for hADR-α1A expression level in the cell models used in this study. As shown in Fig. 6*D*, bioinformatics analysis showed that mRNA expression levels of hADR-α1A are very low in the cell lines used in this study (just above background) and do not correlate with the antiproliferative effects of LLY-507, suggesting that the observed phenotypic effects of LLY-507 are not due to inhibition of hADR-α1A.

## Discussion

In this study, we disclose LLY-507, a small molecule inhibitor of SMYD2 with the following biochemical, biophysical, and cellular properties: 1) has low nanomolar IC_50_ in SMYD2 biochemical assays; 2) has 100-fold selectivity for SMYD2 over 24 other protein or DNA methyltransferases including related family members SMYD3, SUVH420H1, and SUV420H2; 3) inactive (>20 μm) against 454 kinases, 35 G protein-coupled receptors, 14 nuclear hormone receptors, and three cytochrome P450 enzymes; 4) binds to substrate channel of SMYD2 based upon a high (1.6-Å) resolution crystal structure; 5) has submicromolar cell-based potency as demonstrated by its ability to inhibit the mono-methylation of Lys^370^ of p53 in several different cell systems; and 6) has antiproliferative activity in tumor types in which SMYD2 is amplified and/or overexpressed. A small molecule inhibitor of SMYD2 was previously disclosed which, although selective and potent against recombinant SMYD2 enzyme, showed weak cellular activity (25). LLY-507 represents the first cell-potent, selective small molecule inhibitor for SMYD2.

Having a small molecule probe as a tool to investigate SMYD2 biology has advantages over other methods for regulating SMYD2 activity in cells such as CRISPR or siRNA/shRNA-based approaches. First, genetic knock-outs/knockdowns in general are resource- and time-consuming, require optimization of transfection and/or selection conditions, and are often compounded by compensatory changes by the cell due to lethality, making it difficult to make conclusions on whether the observed biology is due to compensation or directly due to knock-out/knockdown of the target. In contrast, working with chemical inhibitors is technically much less challenging as compounds spanning a concentration range are added directly to cells and act over a much shorter time span, leaving little opportunity for the cell to compensate. Second, a small molecule probe provides the ability to study target catalytic function without having to completely eliminate the protein or disrupt contacts it may have with potential binding partners. Third, precise measurements of cellular functional potency and temporal kinetics as we performed in this study (Figs. 3*D* and 6*A*) are readily feasible with small molecule probes in contrast to genetic knock-out/knockdowns, which are often performed at a single concentration and time point optimized for maximum protein elimination.

A PubMed search revealed a modest number of publications on SMYD2 since its identification in 2006. Although reports show that SMYD2 methylates key cancer proteins and suggest that this methyltransferase may be implicated in cancer, they have not as a whole revealed a driver substrate and/or mechanism of SMYD2 oncogenesis. A chemical tool such as LLY-507 enables further probing of this biology, and preliminary investigation using LLY-507 in this study has already started to bear fruit. For example, profiling of LLY-507 in multiple panels of cancer cell lines in both standard and longer duration proliferation studies revealed that 1) the catalytic function of SMYD2 plays a role not only in ESCC but in HCC and breast cancer cell proliferation and that 2) the sensitivity of tumor cell proliferation to inhibition of SMYD2 catalytic function does not depend on levels of SMYD2 expression or the methylation of either p53 or Rb. The lack of a driver role for p53 in SMYD2-mediated tumor cell proliferation is consistent with observations by Komatsu *et al.* (27) depicting that 1) although overall survival rates were significantly worse for SMYD2-positive compared to SMYD2-negative p53-negative ESCC patients, no significant difference in overall survival was noted between SMYD2-positive and SMYD2-negative patients in the p53-positive group, and 2) the inhibition of ESCC cell proliferation with SMYD2 knockdown was independent of p53. Based on the proliferation data in this study, it is plausible that the mechanisms by which SMYD2 drives tumor cell proliferation may involve more than one substrate and/or a second genetic or epigenetic alteration, and the mechanism(s) of SMYD2 oncogenesis may differ depending on cell type. In-depth investigation of the various substrates of SMYD2 in tumor cell proliferation may now be facilitated using the molecular probe LLY-507. Identification of such substrates may provide a better understanding of the role that SMYD2 plays during oncogenesis.

Also novel to this study is the finding that, in contrast to histone methyltransferases such as EZH2, DOT1L, and G9a, SMYD2 does not affect the methylation of any of the predominant H3 or H4 lysine residues at a genome-wide level in cells as revealed from mass spectrometry proteomic analysis of histones derived from LLY-507-treated, SMYD2-overexpressing KYSE-150 cells. This is consistent with work by Diehl *et al.* (32) showing that targeted deletion of SMYD2 in cardiomyocytes does not affect global levels of mono-, di-, or trimethylation of either H3K4 or H3K36 in cell-derived histones as measured by Western blotting. This may be explained by the observation in this study of a largely cytoplasmic localization of endogenous SMYD2 protein in KYSE-150 ESCC cells. Also in line with these results is the finding by Donlin *et al.* (19) that following transfection in cells only 20% of SMYD2 is found in the nucleus, whereas the remaining 80% is in the cytoplasm. These results suggest that a significant proportion of the biological activity exerted by SMYD2 may be occurring in the cytoplasm.

Analysis of the selectivity profile of LLY-507 as well as of the previously disclosed AZ-505 (25) shows that selectivity of small molecules to the SMYD family can be achieved as demonstrated with the >100-fold selectivity of LLY-507 over related family members SMYD3, SUV420H1, and SUV420H2. The high resolution structure of SMYD2 in complex with LLY-507 reveals the significant overlap of the ligand binding site with the substrate peptide binding site and provides a plausible rationale for the selectivity of LLY-507 for SMYD2. In conclusion, the results in this study show that LLY-507 is a cell-active and selective inhibitor of SMYD2, and serves as a valuable chemical probe to elucidate the role of SMYD2 in cancer and other biological processes.

## Supplementary Material

Supplemental Data

## Footnotes

2 The abbreviations used are:

SET: Su(var)3–9, enhancer of zeste, and trithorax
ESCC: esophageal squamous cell carcinoma
HCC: hepatocellular carcinoma
Rb: retinoblastoma
SMYD: SET and MYND
MYND: myeloid translocation protein-8, Nervy, and DEAF-1
LLY-507: 3-cyano-5-{2-[4-[2-(3-methylindol-1-yl)ethyl]piperazin-1-yl]-phenyl}-*N*-[(3-pyrrolidin-1-yl)propyl]benzamide
me1: mono-methylated
RIPA: radioimmunoprecipitation assay.
