# Supplementary material for: LLY-507, a Cell-active, Potent, and Selective Inhibitor of Protein-lysine Methyltransferase SMYD2
Source: J Biol Chem. 2015 Mar 30;290(22):13641–53. doi: 10.1074/jbc.M114.626861 (PMC4447944; doi:10.1074/jbc.M114.626861)
Supplement: Supplemental Data [file supp_290_22_13641__index.html]

LLY-507, a Cell-Active, Potent and Selective Inhibitor of Protein Lysine Methyltransferase SMYD2 — LLY-507, a Cell-active, Potent, and Selective Inhibitor of Protein-lysine Methyltransferase SMYD2 — Characterization of SMYD2 Small Molecule Inhibitor LLY-507 — Supplemental Data 

# LLY-507, a Cell-active, Potent, and Selective Inhibitor of Protein-lysine Methyltransferase SMYD2

## Supplemental Data

**Files in this Data Supplement:**

- Supplemental (.pdf, 807 KB) - SUPPLEMENTAL TABLE 1: Summary of biochemical assay conditions for the methyltransferase selectivity panel. SUPPLEMENTAL TABLE 2: Effect of LLY-507 on the activity of 454 human kinases. SUPPLEMENTAL TABLE 3: Effect of LLY-507 on the activity of 36 G protein-coupled receptors, using the Eurofins-CEREP pharmacology platform. SUPPLEMENTAL TABLE 4: Effect of LLY-507 against 15 nuclear hormone receptors and 3 cytochrome p450 enzymes, using the Eurofins-CEREP pharmacology platform. SUPPLEMENTAL FIGURE 5: Effect of LLY-507 on cellular post-translational modifications on histones H3 and H4 following treatment with LLY-507, as measured by mass spectrometry. SUPPLEMENTAL METHODS: Chemical synthesis of LLY-507
