# Supplementary material for: LLY-507, a Cell-active, Potent, and Selective Inhibitor of Protein-lysine Methyltransferase SMYD2
Source: J Biol Chem. 2015 Mar 30;290(22):13641–53. doi: 10.1074/jbc.M114.626861 (PMC4447944; doi:10.1074/jbc.M114.626861)
Supplement: Supplemental Data [file supp_M114.626861_jbc.M114.626861-1.pdf]

SUPPLEMENTAL INFORMATION

LLY-507, a Cell-Active, Potent and Selective Inhibitor of Protein Lysine Methyltransferase SMYD2

Hannah Nguyen, Abdellah Allali-Hassani, Stephen Antonysamy, Shawn Chang, Lisa Hong Chen, Carmen Curtis, Spencer Emtage, Li Fan, Tarun Gheyi, Fengling Li, Shichong Liu, Joseph R. Martin, David Mendel, Jonathan B. Olsen, Laura Pelletier, Tatiana Shatseva, Song Wu, Feiyu Fred Zhang, Cheryl H. Arrowsmith, Peter J. Brown, Robert M. Campbell, Benjamin A. Garcia, Dalia Barsyte-Lovejoy, Mary Mader and Masoud Vedadi

SUPPLEMENTAL TABLE 1: Summary of biochemical assay conditions for the methyltransferase selectivity panel.

Protein		Substrate (μM) peptide/ nucleosome	Substrate (μM) SAM	pH	Buffer	DTT (mM)	TCEP (mM)	Triton-X100 (%)	Enzyme (nM)
**PRMT1**		0.13	4.6	8	Tris-HCl	5	0	0.01	15
**PRMT3**		0.57	28.3	8	Tris-HCl	5	0	0.01	20
**PRMT6**		0.6	2.3	8	Tris-HCl	5	0	0.01	50
**PRMT8**		0.7	2.2	8	Tris-HCl	5	0	0.01	20
**G9a**		0.8	8	8	potassium phosphate	0	0	0.01	5
**EHMT1 (GLP)**		0.6	8	8	potassium phosphate	0	0	0.01	5
**SUV39H2**		0.5	2.6	8	potassium phosphate	0	0	0.01	10
**SETDB1**		*1.7*	15	8	Tris-HCl	5	0	0.01	10
**SETD2**		7.5	1	9	Tris-HCl	5	0	0.01	500
**SETD7**		2	2	8	Tris-HCl	5	0	0.01	20
**SETD8**		40	60	8	Tris-HCl	0	2	0.01	50
**DOT1L**		1	1	8	Tris-HCl	5	0	0.01	10
**SUV420H1**		2.8	12.5	8	Tris-HCl	5	0	0.01	100
**SUV420H2**		0.9	9	8	Tris-HCl	5	0	0.01	500
**DNMT1**		0.6	2	8	Tris-HCl	5	0	0.01	100
**SMYD2**		3	0.5	9	Tris-HCl	5	0	0.01	30
**PRDM9**		3	140	9	Tris-HCl	5	0	0.01	1
**SMYD3**		1	10	9	Tris-HCl	5	0	0.01	500
**NSD1**		0.2	2	9	Tris-HCl	0	2		20
**NSD2**		0.4	3.6	9	Tris-HCl	0	2	0.01	50
**NSD3**		0.3	3.7	9	Tris-HCl	0	2	0.01	50
**PRMT5-MEP50 Complex**	**PRMT5**	0.12	2	8	Tris-HCl	5	0	0.01	15
	**MEP50**								
**MLL1 Complex**	**MLL1**	2	2	8	Tris-HCl	5	0	0.01	20
	**ASH2L**								
	**RBBP5**								
	**WDR5**								
**MLL3 Complex**	**MLL3**	12	55	9	Tris-HCl	5	0	0.01	100
	**ASH2L**								
	**RBBP5**								
	**WDR5**								
**EZH1 Complex**	**EED**	1	4	8	Tris-HCl	5	0	0.01	10
	**EZH1**								
	**SUZ12**								
	**RBBP4**								
	**AEBP2**								
**EZH2 Complex**	**EED**	1	2	8	Tris-HCl	5	0	0.01	20
	**EZH2**								
	**SUZ12**								

SUPPLEMENTAL TABLE 2: Effect of LLY-507 on the activity of 454 human kinases. LLY-507 was profiled for kinase inhibitory activity using the DiscoverX KINOMEscan℠ Assay platform. *Inhibition was <50% at the 20 μM dose.

Kinase Target	IC50 (μM)	Kinase Target	IC50 (μM)	Kinase Target	IC50 (μM)
hABL1	>20.0	hAURKA	>20.0	hCLK3	>20.0
hABL1(F317I)	>20.0	hAURKB	>20.0	hCLK4	>20.0
hABL1(F317L)	>20.0	hAURKC	>20.0	hCSF1R	>20.0
hABL1(H396P)	>20.0	hAXL	>20.0	hCSF1R-autoinhib	7.5*
hABL1(pE255K)	>20.0	bacPKNB	>20.0	hCSK	>20.0
hABL1(pF317I)	>20.0	hBIKE	>20.0	hCSNK1A1	>20.0
hABL1(pF317L)	>20.0	hBLK	>20.0	hCSNK1A1L	>20.0
hABL1(pH396P)	>20.0	hBMPR1A	>20.0	hCSNK1D	>20.0
hABL1(pM351T)	>20.0	hBMPR1B	>20.0	hCSNK1E	>20.0
hABL1(pQ252H)	>20.0	hBMPR2	>20.0	hCSNK1G1	>20.0
hABL1(pT315I)	>20.0	hBMX	>20.0	hCSNK1G2	>20.0
hABL1(pY253F)	>20.0	hBRAF	>20.0	hCSNK1G3	>20.0
hABL1(Q252H)	>20.0	hBRAF(V600E)	>20.0	hCSNK2A1	>20.0
hABL1(T315I)	>20.0	hBRSK1	>20.0	hCSNK2A2	>20.0
hABL1-p	>20.0	hBRSK2	>20.0	hCTK	>20.0
hABL2	>20.0	hBTK	>20.0	hDAPK1	>20.0
hACVR1	>20.0	hBUB1	>20.0	hDAPK2	>20.0
hACVR1B	>20.0	hCAMK1	>20.0	hDAPK3	>20.0
hACVR2A	>20.0	hCAMK1D	>20.0	hDCAMKL1	>20.0
hACVR2B	>20.0	hCAMK1G	>20.0	hDCAMKL2	>20.0
hACVRL1	>20.0	hCAMK2A	>20.0	hDCAMKL3	>20.0
hADCK3	>20.0	hCAMK2B	>20.0	hDDR1	>20.0
hADCK4	>20.0	hCAMK2D	>20.0	hDDR2	>20.0
hAKT1	>20.0	hCAMK2G	>20.0	hDLK	>20.0
hAKT2	>20.0	hCAMK4	>20.0	hDMPK	>20.0
hAKT3	>20.0	hCAMKK1	>20.0	hDMPK2	>20.0
hALK	>20.0	hCAMKK2	>20.0	hDRAK1	>20.0
hALK(C1156Y)	>20.0	hCDKL2	>20.0	hDRAK2	>20.0
hALK(L1196M)	>20.0	hCDKL3	>20.0	hDYRK1A	>20.0
hAMPK-alpha1	>20.0	hCDKL5	>20.0	hDYRK1B	14.5*
hAMPK-alpha2	>20.0	hCHEK1	>20.0	hDYRK2	>20.0
hANKK1	>20.0	hCHEK2	>20.0	hEGFR	>20.0
hARK5	>20.0	hCIT	>20.0	hEGFR(E746-A750del)	>20.0
hASK1	>20.0	hCLK1	>20.0	hEGFR(G719C)	>20.0
hASK2	>20.0	hCLK2	>20.0	hEGFR(G719S)	>20.0

SUPPLEMENTAL TABLE 2 (continued)

Kinase Target	IC50 (µM)	Kinase Target	IC50 (µM)
hEGFR(L747-E749del, A750P)	>20.0	hFGFR1	>20.0
hEGFR(L747-S752del, P753S)	>20.0	hFGFR2	>20.0
hEGFR(L747-T751del,Sins)	>20.0	hFGFR3	>20.0
hEGFR(L858R)	>20.0	hFGFR3(G697C)	>20.0
hEGFR(L858R,T790M)	>20.0	hFGFR4	>20.0
hEGFR(L861Q)	>20.0	hFGR	>20.0
hEGFR(S752-I759del)	>20.0	hFLT1	>20.0
hEGFR(T790M)	>20.0	hFLT3	>20.0
hEIF2AK1	>20.0	hFLT3(D835H)	>20.0
hEPHA1	>20.0	hFLT3(D835Y)	>20.0
hEPHA2	>20.0	hFLT3(ITD)	>20.0
hEPHA3	>20.0	hFLT3(K663Q)	>20.0
hEPHA4	>20.0	hFLT3(N841I)	>20.0
hEPHA5	>20.0	hFLT3(R834Q)	>20.0
hEPHA6	>20.0	hFLT3-autoinhibited	>20.0
hEPHA7	>20.0	hFLT4	>20.0
hEPHA8	>20.0	hFRK	>20.0
hEPHB1	>20.0	hFYN	>20.0
hEPHB2	>20.0	hGAK	>20.0
hEPHB3	>20.0	hGCN2(Kin.Dom.2,S808G)	>20.0
hEPHB4	>20.0	hGRK1	>20.0
hEPHB6	>20.0	hGRK4	>20.0
hERBB2	>20.0	hGRK7	>20.0
hERBB3	>20.0	hGSK3A	>20.0
hERBB4	>20.0	hGSK3B	>20.0
hERK1	>20.0	hHASPIN	>20.0
hERK2	>20.0	hHCK	>20.0
hERK3	>20.0	hHIPK1	>20.0
hERK4	>20.0	hHIPK2	>20.0
hERK5	>20.0	hHIPK3	>20.0
hERK8	>20.0	hHIPK4	>20.0
hERN1	>20.0	hHPK1	>20.0
hFAK	>20.0	hHUNK	>20.0
hFER	>20.0	hICK	>20.0
hFES	>20.0	hIGF1R	>20.0

SUPPLEMENTAL TABLE 2 (continued)

Kinase Target	IC50 (µM)	Kinase Target	IC50 (µM)
hIKK-alpha	>20.0	hLYN	>20.0
hIKK-beta	>20.0	hLZK	>20.0
hIKK-epsilon	>20.0	hMAK	>20.0
hINSR	>20.0	hMAP3K1	>20.0
hINSRR	>20.0	hMAP3K15	>20.0
hIRAK1	>20.0	hMAP3K2	>20.0
hIRAK3	>20.0	hMAP3K3	>20.0
hIRAK4	>20.0	hMAP3K4	>20.0
hITK	>20.0	hMAP4K2	>20.0
hJAK1(JH1Dom.-catalytic)	>20.0	hMAP4K3	>20.0
hJAK1(JH2Dom.-pseudokinase)	>20.0	hMAP4K4	>20.0
hJAK2(JH1Dom.-catalytic)	>20.0	hMAP4K5	>20.0
hJAK3(JH1Dom.-catalytic)	>20.0	hMAPKAPK2	>20.0
hJNK1	>20.0	hMAPKAPK5	>20.0
hJNK2	>20.0	hMARK1	>20.0
hJNK3	>20.0	hMARK2	>20.0
hKIT	>20.0	hMARK3	>20.0
hKIT(A829P)	>20.0	hMARK4	>20.0
hKIT(D816H)	>20.0	hMAST1	>20.0
hKIT(D816V)	>20.0	hMEK1	>20.0
hKIT(L576P)	>20.0	hMEK2	>20.0
hKIT(V559D)	>20.0	hMEK3	>20.0
hKIT(V559D,T670I)	>20.0	hMEK4	>20.0
hKIT(V559D,V654A)	>20.0	hMEK5	>20.0
hKIT-autoinhibited	>20.0	hMEK6	>20.0
hLATS1	>20.0	hMELK	>20.0
hLATS2	>20.0	hMERTK	>20.0
hLCK	>20.0	hMET	>20.0
hLIMK1	>20.0	hMET(M1250T)	>20.0
hLIMK2	>20.0	hMET(Y1235D)	>20.0
hLKB1	>20.0	hMINK	>20.0
hLOK	>20.0	hMKK7	>20.0
hLRRK2	>20.0	hMKNK1	>20.0
hLRRK2(G2019S)	>20.0	hMKNK2	>20.0
hLTK	>20.0	hMLCK	>20.0

SUPPLEMENTAL TABLE 2 (continued)

Kinase Target	IC50 (µM)	Kinase Target	IC50 (µM)	Kinase Target	IC50 (µM)
hMLK1	>20.0	hp38-delta	>20.0	hPIK3CA(Q546K)	>20.0
hMLK2	>20.0	hp38-gamma	>20.0	hPIK3CB	>20.0
hMLK3	>20.0	hPAK1	>20.0	hPIK3CD	>20.0
hMRCKA	>20.0	hPAK2	>20.0	hPIK3CG	>20.0
hMRCKB	>20.0	hPAK3	>20.0	hPIK4CB	>20.0
hMST1	>20.0	hPAK4	>20.0	hPIM1	>20.0
hMST1R	>20.0	hPAK6	>20.0	hPIM2	>20.0
hMST2	>20.0	hPLK4	>20.0	hPIM3	>20.0
hMST3	>20.0	prstePFCDPK1	>20.0	hPIP5K1A	16.4
hMST4	>20.0	prstePFPK5	>20.0	hPIP5K1C	>20.0
hMTOR	>20.0	hPRKCD	>20.0	hPIP5K2B	>20.0
hMUSK	>20.0	hPRKCE	>20.0	hPIP5K2C	>20.0
hMYLK	>20.0	hPRKCH	>20.0	hPKAC-alpha	>20.0
hMYLK2	>20.0	hPAK7	>20.0	hPKAC-beta	>20.0
hMYLK4	>20.0	hPCTK1	>20.0	hPKMYT1	>20.0
hMYO3A	>20.0	hPCTK2	>20.0	hPKN1	>20.0
hMYO3B	>20.0	hPCTK3	>20.0	hPKN2	>20.0
hNDR1	>20.0	hPDGFRA	>20.0	hPLK1	>20.0
hNDR2	>20.0	hPDGFRB	>20.0	hPLK2	>20.0
hNEK1	>20.0	hPDPK1	>20.0	hPLK3	>20.0
hNEK10	>20.0	hPFTAIRE2	>20.0	hPLK4	>20.0
hNEK11	>20.0	hPFTK1	>20.0	prstePFCDPK1	>20.0
hNEK2	>20.0	hPHKG1	>20.0	prstePFPK5	>20.0
hNEK3	>20.0	hPHKG2	>20.0	hPRKCD	>20.0
hNEK4	>20.0	hPIK3C2B	>20.0	hPRKCE	>20.0
hNEK5	>20.0	hPIK3C2G	>20.0	hPRKCH	>20.0
hNEK6	>20.0	hPIK3CA	>20.0	hPRKCI	>20.0
hNEK7	>20.0	hPIK3CA(C420R)	>20.0	hPRKCQ	>20.0
hNEK9	>20.0	hPIK3CA(E542K)	>20.0	hPRKD1	>20.0
hNIK	>20.0	hPIK3CA(E545A)	>20.0	hPRKD2	>20.0
hNIM1	>20.0	hPIK3CA(E545K)	>20.0	hPRKD3	>20.0
hNLK	>20.0	hPIK3CA(H1047L)	>20.0	hPRKG1	>20.0
hOSR1	>20.0	hPIK3CA(H1047Y)	>20.0	hPRKG2	>20.0
hp38-alpha	>20.0	hPIK3CA(I800L)	>20.0	hPRKR	>20.0
hp38-beta	>20.0	hPIK3CA(M1043I)	>20.0	hPRKX	>20.0

SUPPLEMENTAL TABLE 2 (continued)

Kinase Target	IC50 (μM)	Kinase Target	IC50 (μM)
hPRP4	>20.0	hSGK3	>20.0
hPYK2	>20.0	hSIK	>20.0
hQSK	>20.0	hSIK2	>20.0
hRAF1	>20.0	hSLK	>20.0
hRET	>20.0	hSNARK	>20.0
hRET(M918T)	>20.0	hSNRK	>20.0
hRET(V804L)	>20.0	hSRC	>20.0
hRET(V804M)	>20.0	hSRMS	>20.0
hRIOK1	>20.0	hSRPK1	>20.0
hRIOK2	>20.0	hSRPK2	>20.0
hRIOK3	>20.0	hSRPK3	>20.0
hRIPK1	>20.0	hSTK16	>20.0
hRIPK2	>20.0	hSTK33	>20.0
hRIPK4	>20.0	hSTK35	>20.0
hRIPK5	>20.0	hSTK36	>20.0
hROCK1	>20.0	hSTK39	>20.0
hROCK2	>20.0	hSYK	>20.0
hROS1	>20.0	hTAK1	>20.0
hRPS6KA4(Kin.Dom.1-N-term)	>20.0	hTAOK1	>20.0
hRPS6KA4(Kin.Dom.2-C-term)	>20.0	hTAOK2	>20.0
hRPS6KA5(Kin.Dom.1-N-term)	>20.0	hTAOK3	>20.0
hRPS6KA5(Kin.Dom.2-C-term)	>20.0	hTBK1	>20.0
hRSK1(Kin.Dom.1-N-term)	>20.0	hTEC	>20.0
hRSK1(Kin.Dom.2-C-term)	>20.0	hTESK1	>20.0
hRSK2(Kin.Dom.1-N-term)	>20.0	hTGFBR1	>20.0
hRSK2(Kin.Dom.2-C-term)	>20.0	hTGFBR2	>20.0
hRSK3(Kin.Dom.1-N-term)	>20.0	hTIE1	>20.0
hRSK3(Kin.Dom.2-C-term)	>20.0	hTIE2	>20.0
hRSK4(Kin.Dom.1-N-term)	>20.0	hTLK1	>20.0
hRSK4(Kin.Dom.2-C-term)	>20.0	hTLK2	>20.0
hS6K1	>20.0	hTNIK	>20.0
hSBK1	>20.0	hTNK1	>20.0
hSGK	>20.0	hTNK2	>20.0
hSgK110	>20.0	hTNNI3K	>20.0
hSGK2	>20.0	hTRKA	>20.0

SUPPLEMENTAL TABLE 2 (continued)

Kinase Target	IC50 (µM)
hTRKB	>20.0
hTRKC	>20.0
hTRPM6	>20.0
hTSSK1B	>20.0
hTTK	>20.0
hTXK	>20.0
hTYK2(JH1Dom.-catalytic)	>20.0
hTYK2(JH2Dom.-pseudokinase)	>20.0
hTYRO3	>20.0
hULK1	>20.0
hULK2	>20.0
hULK3	>20.0
hVEGFR2	>20.0
hVRK2	>20.0
hWEE1	>20.0
hWEE2	>20.0
hWNK1	>20.0
hWNK3	>20.0
hYANK1	>20.0
hYANK2	>20.0
hYANK3	>20.0
hYES	>20.0
hYSK1	>20.0
hYSK4	>20.0
hZAK	>20.0

SUPPLEMENTAL TABLE 3: Effect of LLY-507 on the activity of 36 G protein-coupled receptors, using the Eurofins-CEREP pharmacology platform.

GCPR Target and Assay Mode	% Effect
h5HT2B Calcium Mobilization Agonist	3.4% Stimulation @ 13.3 µM
h5HT2B Calcium Mobilization Antagonist	6.8% Inhibition @ 10 µM
h5HT2B Calcium Mobilization Potentiation	-7.3% Potentiation @ 10 µM
hADORA3 β-arrestin Antagonist	-38.3 Inhibition @ 10 µM
hADORA3 cAMP Agonist	2.6% Stimulation @ 10 µM
hADORA3 cAMP Potentiation	-7.3% Potentiation @ 10 µM
hADRα1A Calcium Mobilization Agonist	-1.9% Stimulation @ 13.3 µM
hADRα1A Calcium Mobilization Antagonist	98% Inhibition @ 10 µM
hADRα1A Calcium Mobilization Potentiation	-7.9% Potentiation @ 10 µM
hADRα2A β-arrestin Antagonist	-27.7% Inhibition @ 10 µM
hADRα2A cAMP Agonist	0.2% Stimulation @ 10 µM
hADRα2A cAMP Potentiation	-20.5% Potentiation @ 10 µM
hADRβ1 β-arrestin Antagonist	-6% Inhibition @ 10 µM
hADRβ1 cAMP Agonist	52.7% Stimulation @ 10 µM
hADRβ1 cAMP Potentiation	32.1% Potentiation @ 10 µM
hADRβ2 β-arrestin Antagonist	-15.6% Inhibition @ 10 µM
hADRβ2 cAMP Agonist	0.1% Stimulation @ 10 µM
hADRβ2 cAMP Potentiation	-27.2% Potentiation @ 10 µM
hD1 β-arrestin Antagonist	43.4% Inhibition @ 10 µM
hD1 cAMP 1% DMSO Potentiation	-19.4% Potentiation @ 10 µM
hD1 cAMP Agonist	-6.8% Stimulation @ 10 µM
hD2L β-arrestin Antagonist	29.8% Inhibition @ 10 µM
hD2L cAMP Agonist	10.1% Stimulation @ 10 µM
hD2L cAMP Potentiation	-27.5% Potentiation @ 10 µM
hH1 β-arrestin Antagonist	30.1% Inhibition @ 10 µM
hH1 Ca Mobilization Agonist	-2.8% Stimulation @ 13.3 µM
hH1 Ca Mobilization Potentiation	-8.5% Potentiation @ 10 µM
hM2 β-arrestin Agonist	5.1% Stimulation @ 10 µM
hM2 β-arrestin Antagonist	36.3% Inhibition @ 10 µM
hM2 β-arrestin Potentiation	-32.2% Potentiation @ 10 µM
hM3 β-arrestin Antagonist	-23.6% Inhibition @ 10 µM
hM3 Calcium Mobilization Agonist	-1.4% Stimulation @ 13.3 µM
hM3 Calcium Mobilization Potentiation	-18% Potentiation @ 10 µM
hOPRm1 β-arrestin Antagonist	-3.9% Inhibition @ 1 µM
hOPRm1 cAMP Agonist	7.4% Stimulation @ 1 µM
hOPRm1 cAMP Potentiation	9.6% Potentiation @ 1 µM

SUPPLEMENTAL TABLE 4: Effect of LLY-507 against 15 nuclear hormone receptors and 3 cytochrome p450 enzymes, using the Eurofins-CEREP pharmacology platform.

Nuclear Hormone Receptors	Relative IC50 (µM)	Cytochrome p450 Enzymes	% inhibition @ 10 µM
hERα	>10	CYP2D6	61.2
hERβ	>10	CYP2C9	34.2
hFXR	>10	CYP3A4	69.1
hLXRα	>10		
hLXRβ	>10		
hPPARα	>10		
hPPARδ	>10		
hPPARγ	>10		
hRARα	>10		
hRARβ	>10		
hRARγ	>10		
hRXRα	>10		
hTRα1	>10		
hTRβ1	>10		
hVDR	>10		

SUPPLEMENTAL FIGURE 5: Effect of LLY-507 on cellular post-translational modifications on histone H3 following treatment with LLY-507, as measured by mass spectrometry. Un, unmethylated; me1, mono-methylated; me2, di-methylated; me3, tri-methylated; ac, acetylated

A

Histone H3	Relative abundance		Fold difference	
	Vehicle	LLY-507, 5 µM	LLY-507/Vehicle	t-test
**Histone H3 Peptide: TKQTAR**				
H3 K4un	85.5 ± 0.9	83.8 ± 1.7	1.0	0.189
H3 K4me1	10.5 ± 0.3	13.0 ± 1.0	0.8	0.077
H3 K4me2	2.6 ± 0.6	2.1 ± 0.4	1.2	0.080
H3 K4me3	1.4 ± 0.3	1.0 ± 0.2	1.3	0.020
**Histone H3 Peptide: KSTGGKAPR**				
H3 K9un, K14un	10.2 ± 0.3	10.9 ± 1.4	0.9	0.496
H3 K9me1, K14un	7.6 ± 1.2	9.7 ± 2.4	0.8	0.374
H3 K9me2, K14un	26.7 ± 0.9	24.8 ± 0.2	1.1	0.043
H3 K9me3, K14un	18.6 ± 0.6	17.4 ± 0.4	1.1	0.019
H3 K9ac, K14un	2.1 ± 0.9	2.5 ± 0.6	0.9	0.697
H3 K9un, K14ac	4.3 ± 0.7	4.5 ± 0.5	1.0	0.700
H3 K9me1, K14ac	6.2 ± 0.7	8.0 ± 0.7	0.8	0.148
H3 K9me2, K14ac	16.6 ± 0.9	15.3 ± 2.8	1.1	0.528
H3 K9me3, K14ac	7.0 ± 0.2	6.3 ± 1.3	1.1	0.453
H3 K9ac, K14ac	0.7 ± 0.1	0.8 ± 0.1	0.9	0.057
**Histone H3 Peptide: KSAPATGGVKKPHR**				
H3 K27un, K36un	6.1 ± 0.9	5.2 ± 0.8	1.2	0.377
H3 K27un, K36me1	2.1 ± 0.3	2.3 ± 0.8	0.9	0.726
H3 K27me1, K36un	9.0 ± 0.6	10.7 ± 1.4	0.8	0.209
H3 K27me2, K36un	26.0 ± 0.8	22.1 ± 1.8	1.2	0.098
H3 K27un, K36me2	5.6 ± 0.05	4.9 ± 0.5	1.1	0.165
H3 K27me3, K36un	7.5 ± 0.3	6.7 ± 0.5	1.1	0.135
H3 K27ac, K36un	0.1 ± 0.01	0.1 ± 0.004	1.0	0.712
H3 K27me1, K36me1	5.1 ± 0.1	6.9 ± 2.3	0.7	0.297
H3 K27me2, K36me1	16.5 ± 0.8	15.2 ± 2.3	1.1	0.423
H3 K27me1, K36me2	11.6 ± 0.5	14.9 ± 1.7	0.8	0.094
H3 K27me2, K36me2	3.4 ± 0.3	3.1 ± 1.1	1.1	0.639
H3 K27me3, K36me1	3.2 ± 0.1	3.3 ± 0.7	1.0	0.823
H3 K27me1, K36me3	3.2 ± 0.1	4.1 ± 0.4	0.8	0.050
H3 K27me3, K36me2	0.6 ± 0.04	0.5 ± 0.2	1.2	0.436
**Histone H3 Peptide: EIAQDFKTDLR**				
H3 K79un	78.6 ± 4.1	74.8 ± 2.3	1.1	0.128
H3 K79me1	16.2 ± 3.3	17.1 ± 0.8	0.9	0.640
H3 K79me2	5.1 ± 0.8	8.1 ± 2.3	0.6	0.105

SUPPLEMENTAL FIGURE 5 (continued): Effect of LLY-507 on cellular post-translational modifications on histone H4 following treatment with LLY-507, as measured by mass spectrometry. Un, unmethylated; me1, mono-methylated; me2, di-methylated; me3, tri-methylated; ac, acetylated

B

Histone H4	Relative abundance		Fold difference	
	Vehicle	LLY-507, 5 µM	LLY-507/vehicle	t-test
**Histone H4 peptide: GKGGKGLGKGGAKR**				
H4 K5un, K8un, K12un, K16un	37.8 ± 5.6	36.4 ± 3.4	1.04	0.778
H4 K5ac, K8un, K12un, K16un	2.9 ± 1.9	2.6 ± 1.6	1.12	0.873
H4 K5un, K8ac, K12un, K16un	7.0 ± 4.8	11.6 ± 2.7	0.61	0.309
H4 K5un, K8un, K12ac, K16un	17.9 ± 7.2	16.7 ± 7.4	1.07	0.859
H4 K5un, K8un, K12un, K16ac	19.2 ± 9.4	18.0 ± 4.5	1.06	0.884
H4 K5ac, K8ac, K12un, K16un	0.5 ± 0.3	0.4 ± 0.1	1.26	0.625
H4 K5ac, K8un, K12ac, K16un	3.0 ± 1.7	1.6 ± 0.7	1.90	0.273
H4 K5ac, K8un, K12un, K16ac	0.6 ± 0.5	1.6 ± 0.7	0.38	0.201
H4 K5un, K8ac, K12ac, K16un	1.5 ± 0.2	1.5 ± 0.2	0.98	0.854
H4 K5un, K8ac, K12un, K16ac	2.9 ± 1.4	2.7 ± 0.6	1.06	0.772
H4 K5un, K8un, K12ac, K16ac	3.7 ± 1.0	4.3 ± 0.2	0.87	0.361
H4 K5ac, K8ac, K12ac, K16un	0.4 ± 0.1	0.3 ± 0.1	1.31	0.199
H4 K5ac, K8ac, K12un, K16ac	0.5 ± 0.2	0.4 ± 0.1	1.11	0.737
H4 K5ac, K8un, K12ac, K16ac	0.4 ± 0.05	0.4 ± 0.1	1.01	0.870
H4 K5un, K8ac, K12ac, K16ac	1.4 ± 0.5	1.2 ± 0.2	1.19	0.381
H4 ac, K8ac, K12ac, K16ac	0.2 ± 0.1	0.3 ± 0.1	0.70	0.544
**Histone H4 Peptide: KVLR**				
H4 K20un	10.0 ± 0.9	10.2 ± 2.3	0.98	0.907
H4 K20me1	27.4 ± 8.0	31.0 ± 5.5	0.88	0.627
H4 K20me2	61.8 ± 8.8	58.0 ± 7.7	1.07	0.669
H4 K20me3	0.8 ± 0.1	0.8 ± 0.1	0.97	0.796

SUPPLEMENTAL METHODS

Chemical synthesis of LLY-507

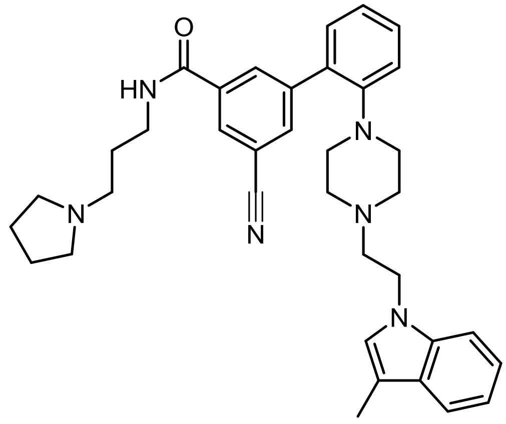

Route to LLY-507

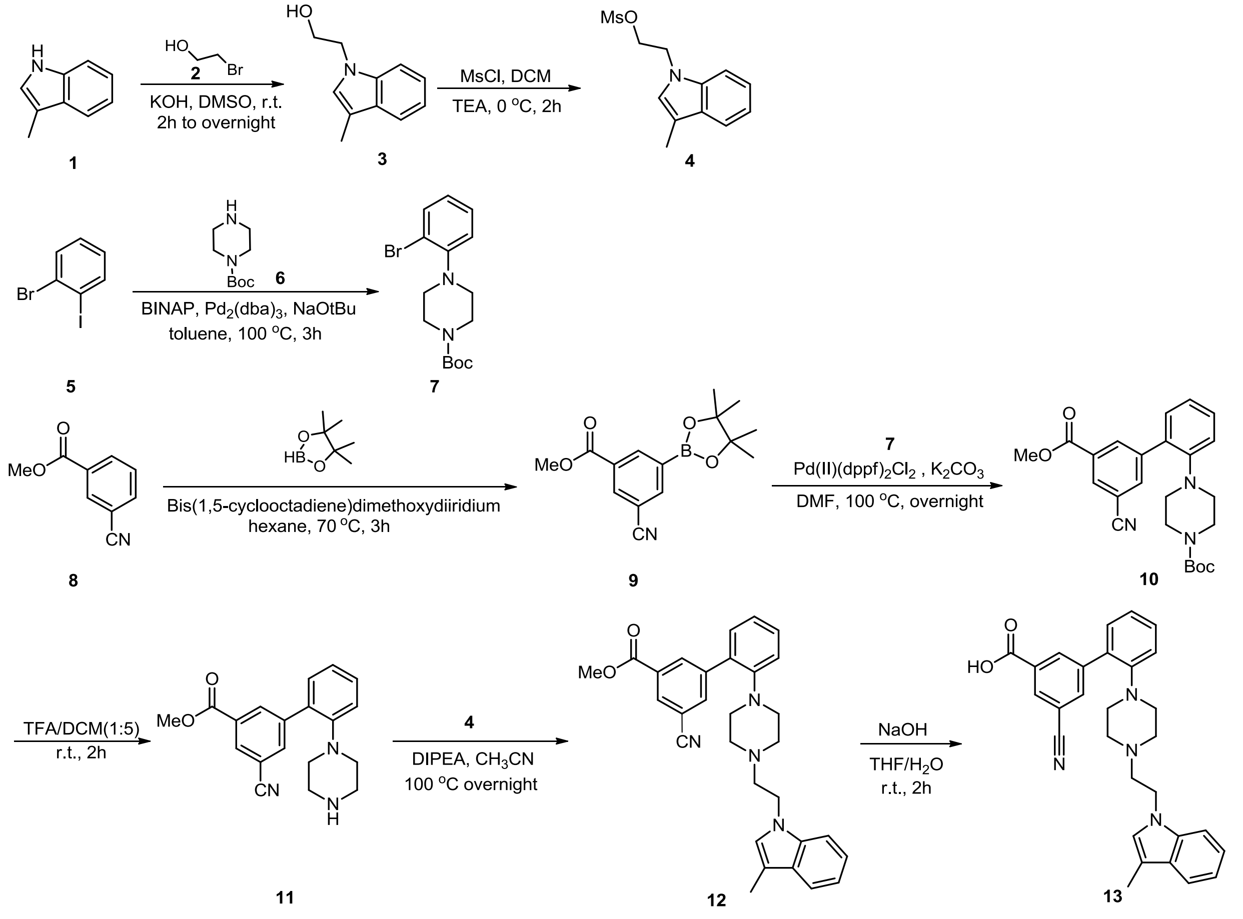

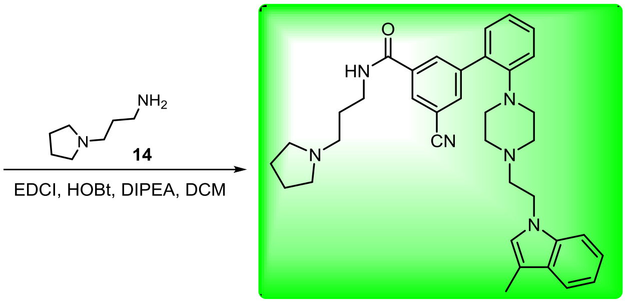

LLY-507

Step 1-Preparation of compound 3

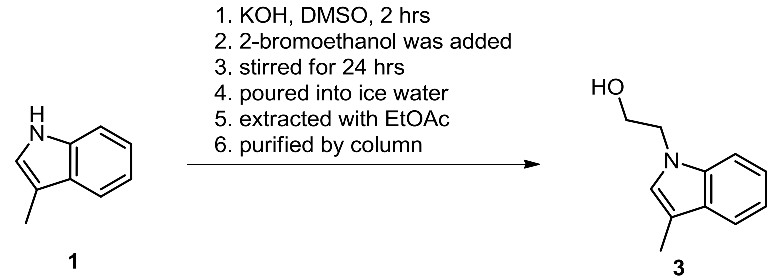

1 **3**

A mixture of 3-methyl-1H-indole (70.00 g, 533.64 mmol) and KOH (205.63 mL, 299.40 g, 5.34 mol) in DMSO (700 mL) was stirred at 16 °C for 2 hr. Then to the mixture was added 2-bromoethanol (37.83 mL, 66.69 g, 533.64 mmol) in one portion.

The mixture was stirred at 16 °C for 24 hr when TLC showed the reaction was complete.

The reaction mixture was poured into 3.0L of ice/water and extracted with EtOAc (500 mL x 3). The combined organic phase was concentrated and purified by column (petroleum ether/ EtOAc = 5/1 to 3/1) to give 2-(3-methylindol-1-yl)ethanol (46.50 g, 265.37 mmol; 49.73% yield) as brown slurry.

Step 2-Preparation of compound 4

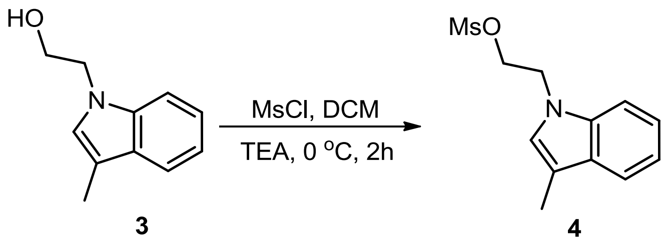

At 0 °C, to a mixture of 2-(3-methylindol-1-yl)ethanol (46.00 g, 262.52 mmol) and triethylamine (73.08 mL, 53.13 g, 525.03 mmol) in dichloromethane (600 mL) was added methanesulfonyl chloride (24.38 mL, 36.09 g, 315.02 mmol) dropwise.

The mixture was stirred at room temperature for 2 hrs.

LCMS showed the reaction was complete. The mixture was poured into 100 mL of ice water, extracted with dichloromethane (100 mL x 2).

The combined organic phase was dried over Na_2SO_4, and concentrated to give the crude product2-(3-methylindol-1-yl)ethyl methanesulfonate (55.00 g, 217.12 mmol; 82.70% yield), which was used for next step directly.

Step 3-Preparation of compound 7

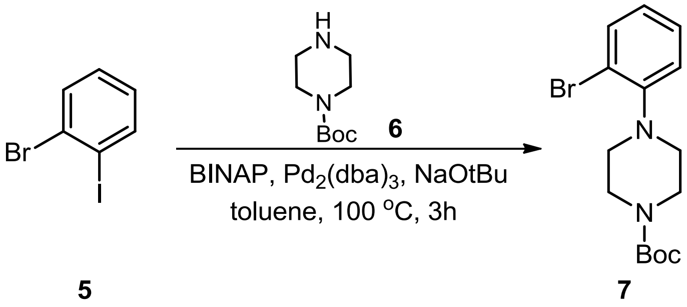

A mixture of 1-bromo-2-iodo-benzene (90.91 mL, 200.00 g, 706.95 mmol) and tert-butyl piperazine-1-carboxylate (158.00 g, 848.34 mmol) and sodium 2-methylpropan-2-olate (101.91 g, 1.06 mol) and (1E,4E)-1,5-diphenylpenta-1,4-dien-3-one;palladium (32.37 g, 35.35 mmol) and (±)-2,2'-

Bis(diphenylphosphino)-1,1'-binaphthalene (44.02 g, 70.70 mmol) in toluene (1500 mL) was stirred at 100 ^{O}C under N_2 for 4 hrs. TLC showed the reaction was complete.

The mixture was diluted with EtOAc and water then filtered. The filtrate was extracted with EtOAc and concentrated. The residue was purified with column to give tert-butyl 4-(2-bromophenyl)piperazine-1-carboxylate as a yellowish slurry.

Another lot from 150 g of 1-bromo-2-iodo-benzene has been run separately, which was worked up together with the first batch. Total yield 120.00 g, 351.66 mmol; was obtained with 28.4% yield.

Step 4-Preparation of compound 9

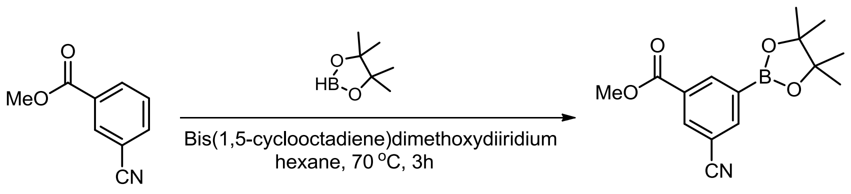

A mixture of methyl 3-cyanobenzoate (100.00 g, 620.51 mmol) 4,4,5,5-tetramethyl-1,3,2-dioxaborolane (87.35 g, 682.56 mmol) (1Z,5Z)-cycloocta-1,5-diene;2,4-dimethyl-BLAHbicyclo[1.1.0]butane (8.23 g, 12.41 mmol) 4-tert-butyl-2-(4-tert-butyl-2-pyridyl)pyridine (5.00 g, 18.62 mmol) in hexane (750 mL) was stirred at 70 °C under N_2 protection for 3 hrs. LCMS showed the reaction was complete.

The mixture was poured to a silica bed. Eluted with petroleum: ethyl acetate (5:1) and concentrated to give the product methyl 3-cyano-5-(4,4,5,5-tetramethyl-1,3,2-dioxaborolan-2-yl)benzoate (100.00 g, 348.29 mmol; 56.13% yield) as a white solid.

Step 5-Preparation of compound 10

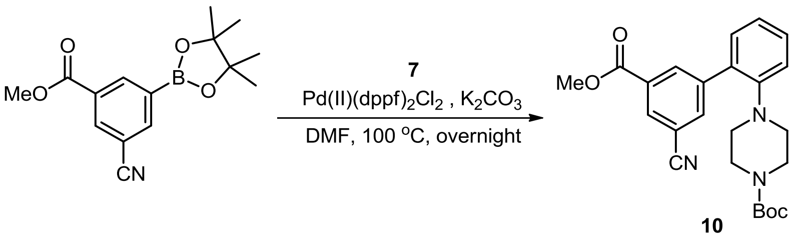

Methyl 3-cyano-5-(4,4,5,5-tetramethyl-1,3,2-dioxaborolan-2-yl)benzoate (100.00 g, 348.29 mmol), tert-butyl 4-(2-bromophenyl)piperazine-1-carboxylate (118.85 g, 348.29 mmol), Pd(dppf)Cl_2 (25.84 g, 34.83 mmol), K_2CO_3 (39.62 mL, 96.27 g, 696.58 mmol) in DMF (1000 mL) were de-gassed and then heated to

80 °C for 4 hrs. TLC (petroleum ether:EtOAc=8:1) showed the starting material was consumed completely. The reaction mixture was poured into H_2O (300 mL). The mixture was extracted with EtOAc (3 x 250 mL). The organic phase was washed with saturated brine (300 mL), dried over anhydrous $MgSO_4$, concentrated in vacuum to give a residue**,** which was pre-purified by column chromatography to afford the pure tert-butyl-4-[2-(3-cyano-5-methoxycarbonyl-phenyl) phenyl]piperazine-1-carboxylate (80.00 g, 189.80 mmol; 54.50% yield) as a slurry.

Step 6-Preparation of compound 11

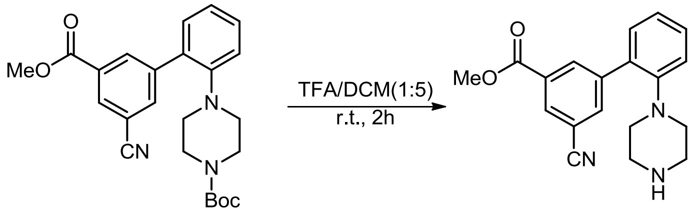

10 **11**

To a mixture of compound **10** (80.00 g, 189.80 mmol) in dichloromethane (1 L) was added TFA (151.49 g, 1.33 mol) in portionwise at r.t. under N_2. The mixture was stirred at r.t. for 16 hrs. TLC showed the reaction was completed. The mixture was poured into ice-water (1000 mL) and adjust with $NaHCO_3$ until pH = 9. The aqueous phase was extracted with dichloromethane (400 mL x 3). The combined organic phase was washed with saturated brine (200 mL x 2), dried with anhydrous Na_2SO_4,filtered and concentrated in vacuum to afford methyl 3-cyano-5-(2-piperazin-1-ylphenyl)benzoate (80.00 g, 211.59 mmol; 111.48% yield) as yellow solid, the crude was used for next step directly.

Step 7-Preparation of compound 12

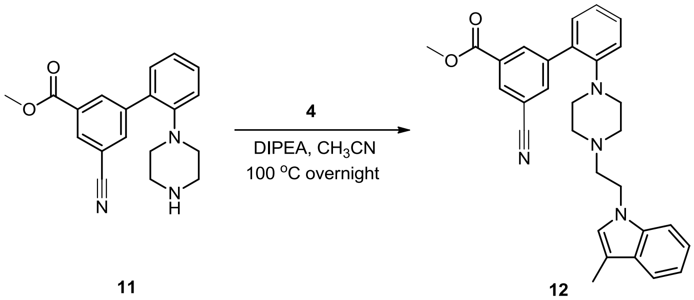

11 **12**

To a mixture of 2-(3-methylindol-1-yl)ethyl methanesulfonate (47.29 g, 186.70 mmol) and methyl 3-cyano-5-(2-piperazin-1-ylphenyl)benzoate (60.00 g, 186.70 mmol) in acetonitrile (200 mL) was added diisopropylethylamine (65.04 mL, 48.26 g, 373.40 mmol) in one portion at r.t.. The mixture was stirred

at 100 °C for 16 hrs. TLC showed the reaction was complete and was then concentrated in vacuum. The residue was purified by silica gel chromatography (column height: 250 mm, diameter: 100 mm, 100-200 mesh silica gel, petroleum ether/ethyl acetate/ dichloromethane = 4/1/1) to afford methyl 3-cyano-5-[2-[4-[2-(3-methylindol-1-yl)ethyl]- piperazin-1-yl]phenyl]benzoate (15.00 g, 31.34 mmol; 16.79% yield) as yellow solid, and 30 g crude product with the purity is ~50%.

Step 8-Preparation of compound 13

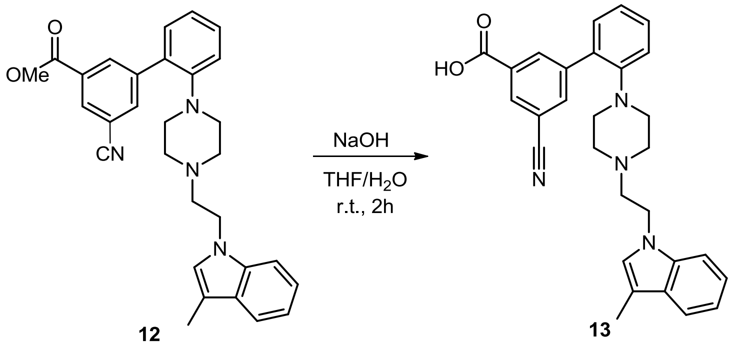

To a mixture of methyl 3-cyano-5-[2-[4-[2-(3-methylindol-1-yl)ethyl] piperazin-1-yl]phenyl]benzoate (15.00 g, 31.34 mmol) in THF (100 mL) was added NaOH (3.76 g, 94.02 mmol) in 50mL of water in one portion at r.t. The mixture was heated to 40 °C and stirred for 2 hrs. TLC showed the reaction was completed.

The mixture was cooled to r.t. and concentrated in reduced pressure at 45 °C. The residue was poured into ice-water (w/w = 1/1) (1500 mL) and adjusted with 2M of HCl to pH=5. The solid formed was dried in vacuo to afford 3-cyano-5-[2-[4-[2-(3-methylindol-1-yl)ethyl]piperazin-1-yl]phenyl] benzoic acid (14.00 g, 30.14 mmol; 96.16% yield) as a yellowish solid, , which was used for next step without purification.

Step 9-Preparation of LLY-507

To a mixture of 5-cyano-2'-(4-(2-(3-methyl-1H-indol-1-yl)ethyl) piperazin-1-yl)-[1,1'-biphenyl]-3-carboxylic acid (15.00 g, 32.29 mmol) and 3-pyrrolidin-1-ylpropan-1-amine (4.14 g, 32.29 mmol) in dichloromethane (20 mL), was added EDCI (7.43 g, 38.75 mmol) and HOBt (5.24 g, 38.75 mmol) and DIPEA (11.25 mL, 8.35 g, 64.58 mmol) in one portion at r.t. The mixture was stirred at r.t. for 16 hrs. HPLC showed the reaction was completed. The mixture was poured into ice-water (150 mL) and extracted with dichloromethane (30 mL x 3). The combined organic phase was washed with saturated brine (50 mL), dried with anhydrous Na_2SO_4, filtered and concentrated in vacuum. The residue was

purified by silica gel chromatography (column height: 250 mm, 100-200 mesh silica gel, dichloromethane/MeOH = 30/1, 10/1) to afford 3-cyano-5-[2-[4-[2-(3-methylindol-1-yl)ethyl]piperazin-1-yl]phenyl]-N-(3-pyrrolidin-1-ylpropyl)benzamide (5.20 g, 9.05 mmol; 28.02% yield) as yellow solid, and some crude product3.0 g with the purity is ~50%.

Note: Due to the high polarity of the product, using DMF as solvent, the yield should be improved significantly.
